# Addressing Heterogeneity in Equine PRP Therapies: A Scoping Review of Methods, Evidence, and Commercial Validation

**DOI:** 10.3390/ani15243586

**Published:** 2025-12-13

**Authors:** Jorge U. Carmona, Catalina López, David Argüelles

**Affiliations:** 1 Grupo de Investigación Terapia Regenerativa, Departamento de Salud Animal, Universidad de Caldas, Calle 65 No 26-10, Manizales 170004, Colombia; 2 Grupo de Investigación Patología Clínica Veterinaria, Departamento de Salud Animal, Universidad de Caldas, Calle 65 No 26-10, Manizales 170004, Colombia; catalina.lopez@ucaldas.edu.co; 3 Department of Animal Medicine and Surgery, Faculty of Veterinary Medicine, Universidad de Córdoba, Campus de Rabanales, 14071 Córdoba, Spain; cu2arcad@uco.es

**Keywords:** equine regenerative medicine, platelet-rich plasma, methodological standardization, centrifugation, commercial PRP kits, leukocyte-rich PRP, leukocyte-poor PRP

## Abstract

(1) Background: Platelet-rich plasma (PRP) therapy has become a cornerstone of equine regenerative medicine, yet significant methodological variability compromises reproducibility and clinical comparability. (2) Methods: This scoping review systematically mapped and analyzed peer-reviewed studies describing equine PRP preparation methods and commercial systems (2000–2024) following PRISMA-ScR guidelines. (3) Results: Twenty-four studies met the inclusion criteria, encompassing 317 horses and both manual and commercial devices. Double-spin manual protocols predominated, though centrifugation parameters, anticoagulants, and activation strategies varied widely. Methodological quality, assessed using an adapted nine-criterion framework, revealed that only 29% of studies achieved comprehensive reporting, particularly lacking platelet-yield and activation details. An additional multilingual web search identified 24 veterinary PRP kits, of which only 10 had published validation in horses, exposing a pronounced gap between marketing claims and scientific evidence. (4) Conclusions: These discrepancies underscore the need for standardized reporting, transparent characterization, and independent evaluation of PRP systems. The proposed framework aims to guide future research toward reproducible, evidence-based practices that enhance therapeutic reliability and clinical translation in equine regenerative medicine.

## Simple Summary

Platelet-rich plasma (PRP) is a treatment derived from a horse’s own blood that is used to help heal injuries. However, the way vets prepare PRP varies greatly, making it difficult to compare results between studies and know which method works best. This review collected and analyzed all the available scientific studies on how PRP is made for horses. We found that many studies do not report their methods completely, making it hard to repeat them. We also discovered that while many commercial PRP preparation kits are sold for horses, less than half have been properly tested and described in scientific publications. This means veterinarians might be using products without solid evidence of how they perform. Our findings highlight the need for clear and consistent reporting in future research. This will help vets choose the most effective treatments, ultimately improving care for injured horses.

## 1. Introduction

Regenerative medicine represents a transformative approach in both human and veterinary medicine that focuses on repairing, replacing, or regenerating damaged tissues and organs to restore their natural function [1,2,3]. Unlike conventional therapies that primarily manage symptoms, regenerative strategies target the root causes of disease by combining the body’s innate healing mechanisms with advanced biotechnological interventions [4,5]. This multidisciplinary field integrates cellular therapies, bioengineering, and molecular tools to promote tissue recovery.

The field employs several innovative approaches to achieve these therapeutic goals [1,2,3]. Stem cell therapies, particularly those utilizing mesenchymal stem cells (MSCs), have shown significant promise for tissue regeneration [6,7]. Growth factor applications, including platelet-rich plasma (PRP) [8,9] and recombinant proteins [10,11], effectively stimulate the body’s natural healing processes. Tissue engineering benefits from biomaterial scaffolds that provide structural support for new tissue growth [1,2], while gene editing technologies such as clustered regularly interspaced short palindromic repeats (CRISPR) offer new possibilities for correcting genetic defects [12].

In veterinary applications, particularly equine medicine, regenerative strategies have been widely adopted [13], particularly for treating orthopedic conditions such as osteoarthritis, tendon and ligament injuries, laminitis, and bone fractures. Additionally, these approaches have been applied in managing distal wounds, extensive burns, and reproductive issues in mares, among other applications [14,15,16].

PRP represents a simple, versatile, and cost-effective regenerative therapy that has gained widespread clinical adoption in equine medicine [13,17,18]. The therapeutic application of PRP in horses emerged in the mid-2000s, when pioneering studies established standardized preparation protocols [19,20] and demonstrated clinical efficacy in managing osteoarthritis [21], tendinopathies, and ligament injuries [22,23]. These initial clinical reports were soon followed by the first [24] in vitro studies characterizing PRP’s anabolic effects on equine tendon and ligament explants [25,26]. Since then, research in this area has expanded significantly, showing a 9.37% annual growth rate and strong academic interest [27].

PRP is fundamentally a plasma-based suspension containing active platelets and leukocytes [28], though its cellular composition varies significantly depending on the preparation protocol [18]. Based on leukocyte content, PRP can be categorized into two main subtypes: leukocyte-poor PRP, also known as pure PRP (P-PRP), which retains minimal or no white blood cells, and leukocyte-rich PRP (L-PRP), which contains elevated concentrations of both platelets and leukocytes [29,30]. Equine P-PRP formulations generally exhibit platelet concentrations similar to or slightly higher than whole blood, whereas L-PRP is distinguished by substantially increased platelet counts alongside detectable leukocyte concentrations [18]. Upon administration, PRP undergoes a transition to a platelet-rich gel (PRG), facilitating the gradual release of bioactive factors such as growth factors (GFs) and immunomodulatory cytokines [29,30]. This mechanism establishes a regenerative microenvironment that recruits endogenous stem cells and activates tissue-repair pathways, thereby supporting the healing of injured structures [18].

Recent systematic reviews evaluating the efficacy of PRP for equine musculoskeletal injuries [31,32,33,34] and endometritis [35] revealed substantial variability across studies in PRP preparation methods (including commercial kits and protocols), reported quality parameters, and procurement documentation. This occurred despite the availability of controlled experimental studies (ECSs), case series (CSs), and randomized clinical trials (RCTs) [31,32,33,35]; similar inconsistencies in human PRP studies [36,37] have prompted calls for standardized reporting to enable more reliable cross-study comparisons [38,39].

Unlike previous systematic reviews that have primarily evaluated the clinical efficacy of PRP in equine musculoskeletal and reproductive disorders [31,32,33,34,35], the present scoping review concentrates on the methodological domain of PRP preparation. Specifically, we analyze the heterogeneity of manual and commercial protocols, identify critical gaps in methodological reporting, and evaluate the degree of scientific validation supporting commercially marketed equine PRP systems. By focusing on preparation methodology rather than clinical outcomes, this review addresses an unmet need in the field and provides a foundation for improving reproducibility, standardization, and translational reliability in equine regenerative medicine.

This scoping review examines the published scientific literature on platelet-rich plasma (PRP) preparation methods in equine regenerative medicine. Our objectives were: (1) to map the methodological heterogeneity in PRP preparation, including both manual protocols and commercial devices; (2) to analyze the degree of methodological transparency and standardization across studies, applying an adapted quality framework; (3) to identify and compare commercial equine PRP preparation kits currently available on the market, distinguishing those supported by peer-reviewed validation from those lacking published scientific evidence, in order to assess the alignment between commercial distribution and evidence-based practice; and (4) to propose minimum reporting guidelines for future veterinary PRP studies, aiming to improve reproducibility, comparability, and clinical translation.

## 2. Materials and Methods

This scoping review did not involve the use of live animals or the implementation of clinical or experimental procedures. As such, it did not require approval from an Animal Ethics Committee. The protocol was not registered.

### 2.1. Protocol for Peer Reviewed Documents

The methodology for this scoping review was developed in accordance with the Preferred Reporting Items for Systematic Reviews and Meta-Analyses extension for Scoping Reviews (PRISMA-ScR) guidelines [40]. A comprehensive literature search was conducted to identify studies describing the methods, techniques, protocols, devices, or commercial kits used to prepare PRP from equine blood. The search was performed without language restriction across three major electronic databases—Web of Science, Scopus, and PubMed—accessed on 14 April 2025, and covered publications from 1 January 2000 to 31 December 2024.

A search strategy was implemented on retrieving documents related to PRP procurement methods, including manual protocols and commercial preparation kits. The search terms for the query were: (equine OR horse) AND (“platelet-rich plasma” OR “platelet concentrates” OR “autologous conditioned plasma” OR “autologous conditioned serum”) AND (techniques OR procedures OR kits).

#### Eligibility Criteria for Publications and Protocol for Data Collection, Screening, and Inclusion

C.L., J.U.C., and D.A independently accessed each database using the predefined keywords for this review. The articles identified were uploaded into Rayyan (Rayyan Systems Inc., Doha, Qatar; accessed 16 April 2025), a free web-based tool designed to support researchers in managing the screening process of systematic and scoping reviews [41]. Each article was independently evaluated based on its title, keywords, and abstract. Discrepancies between reviewers were resolved through consensus discussion.

Articles were considered eligible for inclusion when they provided original data on the preparation of PRP in horses, either through detailed methodological descriptions, validation of commercial kits, or controlled laboratory and clinical reports in which preparation protocols were explicitly described. Only full-text papers were reviewed to ensure that methodological details could be properly assessed.

Studies were excluded if they corresponded to reviews, case reports, abstracts, or conference proceedings; if they were conducted in species other than horses; or if they failed to provide essential methodological information, such as centrifugation parameters in g or relative centrifugation force (RCF) or rpm with rotor longitude, baseline blood counts, or the brand of the kit employed.

The protocol for this scoping review was not prospectively registered. PROSPERO does not accept registrations for scoping reviews outside human health, and no equivalent international registry currently exists for methodological scoping reviews in veterinary medicine.

### 2.2. Data Charting and Integrated Quality Assessment

A standardized Microsoft Excel form (Microsoft Excel 2019 Home Edition (Microsoft Corp., Redmond, WA, USA)) was developed to extract and chart data from each included study. Three reviewers independently performed the extraction, cross-verifying all entries for accuracy and consistency. The use of commercial kits was also documented, along with any evidence of prior validation in peer-reviewed research. Any discrepancies in extraction were resolved through consensus discussions after jointly revisiting the original sources.

For studies that reported cellular counts or soluble mediator concentrations exclusively in graphical format without providing exact numerical values in the text or tables, data estimation was performed. This process involved digitally extracting data points from figures using a web-based plot digitizer tool (WebPlotDigitizer (Version 4.6); Ankit Rohatgi, Austin, TX, USA; https://automeris.io/WebPlotDigitizer); (accessed 16 April 2025). This tool utilizes advanced image processing algorithms to accurately calibrate axes and extract numerical values. The extractions were independently verified by a second reviewer to ensure consistency and minimize error. All values obtained through this method are explicitly indicated as “estimated from figure” in the results and tables to ensure transparency. This approach was solely used to include otherwise unreported but critical quantitative data in our comparative analysis.

To contextualize the rigor and reproducibility of PRP characterization, we conducted a structured methodological quality assessment. The evaluation framework was adapted from the Platelets editorial policy for PRP studies [38], itself derived from the guidance of the Platelet Physiology Subcommittee of the Scientific and Standardization Committee (SSC) of the International Society on Thrombosis and Haemostasis (ISTH) [42].

This framework establishes the minimum reporting requirements to ensure transparency, reproducibility, and cross-study comparability. From the original eleven domains, we consolidated and restructured the criteria into nine core characteristics (C1–C9) (Table 1).

To ensure methodological transparency and reproducibility, the data extraction form and the nine-criterion evaluation framework were pretested by the review team using a pilot sample of three representative studies prior to full data charting. Each criterion (C1–C9) was independently scored by three reviewers on a 0–10 scale, and discrepancies were resolved by consensus. Both mean and total scores were recorded for descriptive analysis. Inter-reviewer agreement for categorical scoring decisions was assessed using Cohen’s kappa (κ) statistic, calculated with a predefined threshold of κ ≥ 0.80 to indicate excellent concordance.

### 2.3. Internet Search for Commercial PRP Kits

In parallel with the literature-based component of this scoping review, a supplementary multilingual internet search was conducted to identify platelet-rich plasma (PRP) preparation kits marketed for veterinary use in horses. The search was performed between 15 and 17 April 2025, using Google^®^ (Google Search Engine [Internet]. Mountain View, CA, USA: Google; 2025. Available from: https://www.google.com) as the primary search engine. Search terms included combinations of “equine PRP kit” translated into English, Spanish, Portuguese, French, German, and Italian (e.g., “kit PRP caballo,” “kit PRP cavalo,” “PRP-Kit Pferd”).

Websites from manufacturers, veterinary distributors, and online catalogs were screened to retrieve the following information: kit name, manufacturer, country of origin, preparation mechanism (manual, closed system, or semi-automated), and advertised indications or clinical claims.

Once the list of kits was compiled, each product was cross-checked for scientific validation by searching PubMed, Scopus, and Web of Science using the following search string: (Kit name) AND (horse OR equine) AND (“platelet-rich plasma”).

For this review, commercial PRP kits were classified using a simple categorical approach. A kit was considered validated when at least one peer-reviewed publication—of any type—documented its use in equine subjects. Conversely, a kit was classified as non-validated when no peer-reviewed studies were identified. This definition was intended solely to distinguish products with any published evidence from those without peer-reviewed documentation, without implying methodological rigor, performance equivalence, or clinical endorsement.

This approach allowed comparison between the landscape of commercially available PRP kits and the evidence base established in peer-reviewed research, with the aim of identifying potential gaps between marketing presence and scientific validation—a key consideration for clinical decision-making in equine regenerative medicine.

Discrepancies in classification or database output were resolved through consensus among the review team.

## 3. Results

### 3.1. Study Selection

**Figure 1:**
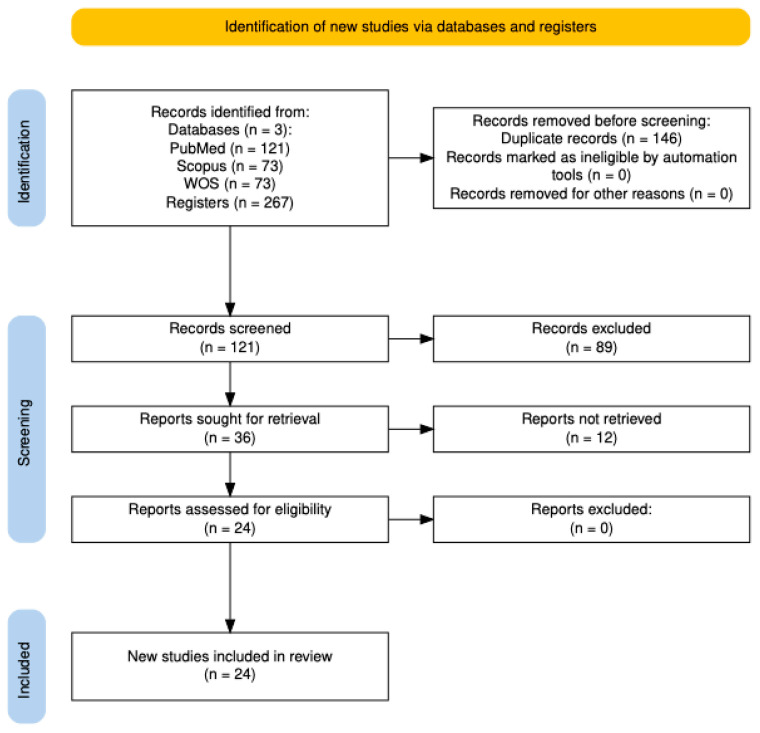
Flow chart of the study according to PRISMSA criteria.

A total of 267 records were initially identified through database searches in PubMed (n = 121), Scopus (n = 73), and Web of Science (n = 73). After removing 146 duplicates, 121 unique records were screened. Following a software-assisted screening of titles and abstracts, 89 records were excluded, leaving 36 reports for full-text review. Of these, twelve were excluded for the following reasons: three did not report adequate centrifugation protocols [43,44,45]; eight provided incomplete information regarding whole blood [46,47,48,49,50,51,52] or PRP cellular composition [53]; and one focused on an autologous protein solution rather than PRP [54]. Consequently, 24 studies [19,20,55,56,57,58,59,60,61,62,63,64,65,66,67,68,69,70,71,72,73,74,75,76] met the inclusion criteria and were included in the scoping review. Among these, 20 were published in English, while the remaining consisted of four in Portuguese [64,65,70,72], one in German [73], and one in Spanish [76]. The complete study selection process is depicted in the PRISMA 2020 flow diagram (Figure 1).

### 3.2. Study Population Characteristics: Sample Size, Breed Distribution, Sex, and Body Weight

Across the 24 included studies, a total of 317 horses were evaluated, with sample sizes ranging from 4 to 40 animals per study [19,20,55,56,57,58,59,60,61,62,63,64,65,66,67,68,69,70,71,72,73,74,75,76,77]. The median sample size was 10 horses, reflecting the typical logistical and ethical constraints of equine clinical research. Breed distribution demonstrated a predominance of athletic and light horse breeds. Mixed-breed and crossbreed horses were the most frequently represented group (appearing in 9 studies) [19,20,55,59,64,71,73,77], followed by Warmbloods (3 studies) [57,69,72] and Thoroughbreds (3 studies) [58,60,62], while Argentinean Creole horses appeared in three studies [63,68,70], and only one study included ponies [67], indicating a research focus on athletic horse populations.

Of the 24 studies analyzed, 12 did not report the sex of the horses [55,56,57,59,60,62,64,71,73,75,76,77]. Of the 12 studies that did, only one used an exclusively female group of 6 mares [65], while four studies used only geldings [19,63,68,74]. Three studies reported a balanced or mixed sex distribution [66,69,72], and the remaining studies showed a clear male predominance. Overall, males accounted for 66.4% of the animals with reported sex.

Age was reported in all 24 studies [19,20,55,56,57,58,59,60,62,63,64,65,66,67,68,69,70,71,72,73,74,75,76,77]. The mean ages ranged from 5 ± 1.5 years [58] to 15 ± 1 years [74]. Fourteen studies reported mean ages between 9 and 14 years [55,56,60,62,63,64,65,66,67,68,69,70,72,73]. Five studies reported mean ages below 7 years [20,58,59,75,77], while three studies reported mean ages above 14 years [65,69,74]. Weight was reported in only 7 of the 24 studies (29%) [56,58,64,67,69,71,74]. The reported weights ranged from 200 kg in a pony study [67] to 543 ± 47 kg in a Warmblood population [69].

### 3.3. Methodological Rigor and Transparency in PRP Characterization

To facilitate a clear and systematic comparison of the extensive methodological data, the 24 included studies [19,20,55,56,57,58,59,60,61,62,63,64,65,66,67,68,69,70,71,72,73,74,75,76], have been organized into two distinct tables. Table S1 consolidates studies utilizing manual preparation methods, while Table S2 focuses on studies that employed or compared commercial systems; this table also includes studies that performed a direct comparison between manual and commercial protocols. Both tables present a consolidated summary of the principal methodological attributes using nine standardized parameters (C1–C9). This structured categorization enables a direct comparison of the key elements influencing PRP characterization, such as anticoagulant choice, centrifugation conditions, cellular composition, growth factor content, and activation strategy.

#### 3.3.1. Blood Source (C1): Autologous Versus Allogeneic Use

All 24 studies evaluated in this review sourced blood exclusively from autologous donors (AUT), as explicitly indicated in each publication [19,20,55,56,57,58,59,60,61,62,63,64,65,66,67,68,69,70,71,72,73,74,75,76]. No study employed allogeneic (ALL) blood sources, and none explored cross-donor applications or immune compatibility issues.

#### 3.3.2. Anticoagulant Use, Blood Volume, and Processing Delay (C2)

Among the 24 studies evaluated, reporting completeness for the C2 characteristic—anticoagulant type, blood volume, blood-to-anticoagulant ratio, and processing delay—was variable. The anticoagulant type was specified in 23 studies (96%) [19,20,56,57,58,59,60,62,63,64,66,67,68,69,70,71,72,73,74,75,76,77], while one study did not report this information for its commercial system [65].

A clear divergence in anticoagulant preference was observed between manual and commercial methods. In manual protocols (Table S1), sodium citrate (SC) was the predominant anticoagulant, used in 11 out of 16 studies (69%) [57,60,61,67,69,70,71,72,73,76,77]. In contrast, commercial systems (Table S2) showed a strong preference for acid citrate dextrose (ACD), which was used in 6 out of 8 studies (75%) [56,58,64,68,75,76]. Citrate phosphate dextrose with adenine (CPDA-1) was used in 4 studies [57,63,67,71], typically in protocols that required larger blood volumes or included cryopreservation steps [67].

Blood volume was reported in 13 studies (54%), with values ranging from 27 mL [73] to 500 mL [56,57,58,60,63,65,67,71,73,75,76,77]. The blood-to-anticoagulant ratio was explicitly stated in 8 studies (33%) [62,63,67,68,69,72,74,76], most commonly adhering to the standard 1:9 proportion. The time interval between blood collection and PRP processing was reported in 10 studies (42%) [58,60,63,64,67,68,69,72,73,76]. In most of these, samples were processed within 30 min, although a few studies described intentional resting periods prior to centrifugation.

#### 3.3.3. PRP Preparation Method (C3): Manual Protocols Versus Commercial Systems

The C3 characteristic, describing the PRP preparation method, was reported in all 24 studies analyzed. The methods were categorized as manual protocols or commercial systems for this review.

Manual protocols were the most prevalent, used in 16 studies (67%) [20,57,59,60,61,62,63,64,66,67,69,70,71,72,73,77] (Table S1). These typically involved open systems requiring manual handling steps such as plasma pipetting or buffy coat aspiration using laboratory centrifuges. The double-spin method was the most frequent approach, generally combining an initial low centrifugal force to separate erythrocytes from plasma, followed by a higher force to concentrate platelets.

Commercial or semi-automated systems were evaluated in 8 studies (33%) [19,56,58,65,68,74,75,76] (Table S1). It is important to note that 4 of these 8 studies (50%) [65,68,75,76] directly compared a commercial system against a manual protocol within their experimental design. The ACP^®^ double-syringe system (Arthrex, Inc., Naples, FL, USA) was the most frequently cited device [58,68,74]. Additional platforms included Proteal^®^ (Bioregenerative Solutions, Barcelona, Spain) [56], E-PET^®^ (Pall Corporation, Port Washington, NY, USA) [65,68], GenesisCS^®^ (Vet-Stem, Inc., Poway, CA, USA) [75], SmartPReP2^®^ (Harvest Technologies, Plymouth, MA, USA) [76], and GPS III^®^ (Biomet Biologics, Warsaw, IN, USA) and Angel^®^ (Arthrex, Inc., Naples, FL, USA) systems within a comparative study [68]. For large-volume processing, the Sequire^®^ kit (PPAI Medical, Ft Myers, FL, USA) together with the Haemonetics Cell Saver 5 apheresis unit was used (Haemonetics Corp., Braintree, MA, USA) [19].

Filtration-based approaches were represented primarily by the E-PET^®^ system, reported in two studies [65,68], which isolates platelets through gravitational filtration rather than centrifugation. Together, these systems illustrate the diversity of commercial PRP technologies, ranging from low-volume, clinician-operated devices (e.g., ACP^®^, Proteal^®^) to high-capacity platforms intended for laboratory-scale processing (e.g., Haemonetics).

The comparative studies [65,68,75,76] were particularly valuable, providing direct insights into differences in platelet yield, leukocyte content, growth factor concentration, and processing time between standardized commercial systems and traditional manual methods.

#### 3.3.4. Centrifugation Protocol (C4): Spin Configuration, g-Force, and Duration

Centrifugation protocols (C4) were explicitly reported in all 24 included studies. Three configurations were identified: single-spin, double-spin, and triple-spin protocols, with double-spin clearly predominating.

Double centrifugation was the most common protocol, used in 17 studies (71%). This method was employed in 13 out of 16 manual studies (81%) [20,57,59,60,61,62,64,67,69,70,71,72,73,77] and in 4 out of 8 commercial studies (50%) [58,65,68,76]. These protocols generally followed a sequential “soft spin–hard spin” design, with first spins between 100–400× *g* for 5–15 min to separate erythrocytes, followed by second spins ranging from 200–2000× *g* for 5–17 min to concentrate platelets.

Single-spin protocols were reported in 6 studies (25%). This approach was less common in manual methods, used in only 2 out of 16 manual studies (12.5%) [57,66] (M2, M3), but was the primary configuration for 4 out of 8 commercial systems (50%) [56,68,74,75] (Angel^®^). These were primarily associated with simplified manual approaches or specific commercial devices designed for rapid processing.

Triple centrifugation was the least common protocol, used in only one study (4%) [63]. This was a manual protocol employing successive separation phases (700× *g*, 1250× *g*, and 2370× *g*) to standardize platelet concentration at a target of 1.0 × 10^6^ PLT/µL.

All 24 studies reported centrifugation force using the standardized metric of relative centrifugal force (×*g*), enabling direct comparison. Of these, five studies [67,69,73,74,77] also reported the speed in revolutions per minute (rpm) but consistently provided the corresponding ×*g* value or a conversion.

Centrifugation temperature control was inconsistently reported, with only 2 studies (8%) specifying centrifugation at 4 °C [59,63], 3 studies (12.5%) at room temperature [19,60,62], and the remaining 19 studies (79%) not specifying temperature conditions.

#### 3.3.5. PRP Extraction Technique (C5): Buffy Coat Harvesting, Direct Plasma Collection, and Closed-System Recovery

The C5 characteristic, focused on the method used to recover the platelet-rich plasma (PRP) fraction after centrifugation, showed significant procedural diversity across the 24 included studies. Three primary approaches were identified:

Manual Buffy Coat Harvesting

This technique was the most prevalent, used in 15 studies (62.5%). It involved the manual aspiration of the leukocyte-platelet-rich buffy coat layer using pipettes or syringes and was predominantly associated with manual double-centrifugation protocols [20,57,59,60,61,62,64,67,69,70,71,72,73,77]. While this method offers flexibility and control over cellular composition, it is highly operator-dependent and introduces a risk of contamination in open-system protocols.

Direct Plasma Collection (Supernatant Aspiration)

This approach was used in 4 studies (17%). It involved harvesting the upper plasma layer following centrifugation, often after a single spin, without specifically targeting the buffy coat [56,66,75]. This technique typically yields a leukocyte-poor PRP (P-PRP) but generally results in a lower platelet concentration compared to buffy coat harvesting.

Closed-System Recovery

This method, exclusive to commercial systems, was employed in 8 studies (33%) [19,56,58,65,68,74,75,76]. These systems integrate blood collection, centrifugation, and PRP extraction into a sealed, proprietary device (e.g., ACP^®^ double-syringe, E-PET^®^ filtration bags, SmartPReP2^®^ automated chamber). This design minimizes manual handling, reduces contamination risk, and standardizes the extraction process. It is important to note that some comparative studies [65,68,75,76] utilized both closed-system recovery for the commercial method and manual techniques for the parallel manual protocol.

Reporting of sterility conditions during extraction was inconsistent. Only 4 studies (17%) explicitly specified processing under sterile laminar flow hoods [63,67,71,75], while the majority did not report the specific conditions, potentially affecting the interpretation of product sterility for clinical applications.

#### 3.3.6. Cellular Composition of Whole Blood and Resulting PRP (C6)

Of the 18 studies included in Tables S1 and S2, 12 (67%) reported both baseline whole blood and resulting PRP values for PLT and WBC [56,57,58,59,60,62,63,64,65,68,75,76]. In contrast, RBC data were reported in only 6 studies [57,62,63,68,72,75].

A differential analysis of the cellular composition reveals distinct patterns based on protocol type and centrifugation strategy. Manual methods consistently demonstrated a superior capacity for high platelet enrichment, often exceeding 4–5× and reaching up to 10× baseline in optimized double-spin protocols [65,76]. However, this high platelet yield was frequently coupled with significant leukocyte incorporation, predominantly resulting in L-PRP products [62,65,69]. Commercial systems yielded more heterogeneous outcomes, with a clear divide between leukocyte-rich systems like GPS III^®^ (5.3× PLT, 6.6× WBC) and E-PET^®^ (3.7× PLT, 1.8× WBC) versus leukocyte-poor systems like ACP^®^ (1.3–1.7× PLT, ~0.1× WBC) and Proteal^®^ (~1.6× PLT, ~0.01× WBC) [56,58,68].

The number of centrifugation steps was a critical determinant of final product composition. Single-spin protocols, whether manual or commercial, typically produced modest platelet enrichment (1.3–2.8×) and were more effective at excluding leukocytes, generally yielding P-PRP [56,57,66]. Double centrifugation was the most common and effective strategy for achieving therapeutic platelet concentrations (typically 4–10×), but it often resulted in L-PRP unless specific buffy-coat avoidance techniques were employed [60,65,71,76]. The single study employing triple centrifugation [63] successfully generated a high-purity, leukocyte-poor PRP (4–5× PLT, minimal WBCs) standardized to a target concentration, indicating this approach may be optimal for achieving high platelet yields with minimal leukocyte contamination.

Whole blood PLT and WBC values fell within expected equine physiological ranges across studies, with PLT counts ranging from 101.8 × 10^3^/µL [62] to 256.5 × 10^3^/µL [60] and WBC values between 5.4 × 10^3^/µL [57] and 9.9 × 10^3^/µL [65]. Only a minority of studies reported RBC content or specified the hematology analyzer used for cell quantification, which introduces methodological variability and limits full comparison of cellular profiles across studies.

#### 3.3.7. Concentration of Soluble Mediators in PRP (C7)

The C7 characteristic refers to the quantification of soluble bioactive mediators in PRP (e.g., growth factors, cytokines), which are crucial for its biological activity. Among the 24 studies on manual and commercial methods detailed in the provided Tables S1 and S2 [19,20,56,57,58,59,60,61,62,63,64,65,66,67,68,69,70,71,72,73,74,75,76,77], 17 (71%) quantified at least one soluble mediator. This indicates that while biochemical profiling is a common practice, a significant portion of the literature (29%) still omits this key characteristic.

The most frequently assessed mediators were transforming growth factor-beta 1 (TGF-β1) and platelet-derived growth factor (PDGF-BB), measured in 9 [19,59,60,63,64,68,69,70,71] and 7 [19,59,63,64,65,68,69] of the 24 studies, respectively. Insulin-like growth factor-1 (IGF-1) was evaluated in only 2 studies [19,76], and vascular endothelial growth factor (VEGF) was quantified in just one [61]. A comprehensive analysis of pro-inflammatory cytokines was not performed in any of these studies. Direct comparison across these studies is challenging due to methodological heterogeneity.

This includes the use of different sample processing methods to release mediators—ranging from physiological activation (e.g., calcium chloride, calcium gluconate, thrombin) to complete cell lysis (e.g., freeze-thaw cycles)—as well as variations in ELISA kits and reporting units (e.g., pg/mL vs. ng/mL).

The analysis of the preparation methods reveals clear trends. Double centrifugation protocols consistently resulted in higher platelet enrichment and subsequent growth factor concentrations compared to single centrifugation. For example, double-spin methods yielded TGF-β_1_ levels as high as 12,400–14,000 pg/mL [60,71], starkly contrasting the more modest outcomes of single-spin protocols, which often showed minimal increase over whole blood values [20,60].

When comparing manual and commercial methods, the growth factor yield is highly system dependent. High-performance commercial systems like GPS^®^ III and E-PET^®^ achieved potent growth factor concentrations (PDGF-BB > 5000 pg/mL, TGF-β_1_ > 1700 pg/mL [65,68]) that rivaled the most effective manual double-spin protocols. Conversely, other commercial kits like ACP^®^, designed to produce pure platelet-rich plasma (P-PRP), resulted in significantly lower growth factor concentrations, highlighting a key trade-off between purity and growth factor potency. Only a few studies [59,60,64,68,70] reported mediator concentrations in both PRP and baseline whole blood, allowing for fold-enrichment calculation.

These studies confirmed higher growth factor levels in PRP, but the magnitude of enrichment varied widely (e.g., >1.5–10× for TGF-β_1_), directly reflecting the heterogeneity in platelet yields and preparation techniques.

#### 3.3.8. Platelet and Leukocyte Yield (C8)

This characteristic reports the fold-increase in platelet (PLT) and leukocyte (WBC) concentration compared to whole blood and the total cell yield. Among the 24 studies analyzed, 18 (75%) reported platelet enrichment ratios, which demonstrated a wide range from 1.2× to 13.1× baseline [19]. The methodology was a primary determinant of this yield: manual double-centrifugation protocols consistently achieved high platelet enrichment (>4×) [59,60,63,64,67,71,76], whereas single-spin commercial systems, particularly those designed to produce pure-PRP (P-PRP) like ACP^®^ and Proteal^®^, resulted in more modest concentrations (1.3–2.0×) [56,58,74].

The total platelet yield, representing the proportion of initial platelets recovered in the final PRP product, was quantitatively reported as a percentage or absolute count in only 7 studies (29%). The yields varied considerably, from 7.5% to 74%, with efficient double-spin manual processes often achieving recoveries greater than 50% [67,77]. In comparison, the total platelet yield from commercial kits frequently fell below this threshold [58,74], highlighting a trade-off between standardization and maximal cell recovery.

The leukocyte yield was directly influenced by the preparation system and protocol intent. Leukocyte-rich PRP (L-PRP), characterized by a WBC fold-increase greater than 1×, was commonly generated using double-spin manual methods or filtration-based commercial systems (i.e., E-PET^®^, GPS III^®^) [59,62,64,65,68]. In contrast, leukocyte-poor PRP (P-PRP), with a WBC yield often significantly below 1×, was consistently produced by specific commercial single-spin devices [56,58,74] and some manual techniques that employed low g-forces or selective extraction to minimize leukocyte inclusion [57,60,73].

#### 3.3.9. Activation Strategy (C9): Physiological Versus Non-Physiological Methods

Of the 24 studies analyzed, 15 reported a method for platelet activation or growth factor release [19,20,59,60,63,64,66,67,68,69,70,72,73,74,76,77], while the remaining 9 did not perform or explicitly state the use of activation [56,57,58,61,62,65,71,75]. Calcium chloride was the most frequently used activating agent, applied in 6 studies [20,64,72,73,76,77]. Freeze-thaw cycles were employed in 5 studies [19,66,67,68,74], and thrombin was used in 2 [64,72]. Calcium gluconate served as an alternative calcium source in 3 studies [64,69,70]. In contrast to these chemical agents, freeze-thaw cycles function by inducing cell lysis rather than physiological activation. Additional non-physiological methods included detergent lysis in 4 studies [63,69,70,76] and extracorporeal shockwave therapy in one study [63].

Regarding preparation methods, activators were used in 10 of the 16 manual studies [20,59,60,63,64,66,67,69,70,72,73,76,77] and in 5 of the 8 commercial studies [19,56,58,68,74]. The nature of activation differed, with manual methods frequently employing physiological activators like calcium compounds, while commercial systems often utilized freeze-thaw cycles for analysis or deferred activation to the in vivo environment. A difference was also observed between centrifugation protocols. Defined activation steps were more common in double centrifugation studies, which often aimed to create high-potency PRP for immediate use [59,60,64,69,70,76,77]. In contrast, single centrifugation protocols, particularly those using commercial kits, more frequently omitted exogenous activation or relied on freeze-thaw cycles for end-point quantification [56,58,66,68,74].

#### 3.3.10. Observations: Methodological Insights, Limitations, and Comparative Efficacy

Of the 24 studies analyzed [19,20,56,57,58,59,60,61,62,63,64,65,66,67,68,69,70,71,72,73,74,75,76,77], the reporting of methodological details was inconsistent, creating significant heterogeneity. While basic procedural steps like autologous blood sourcing and centrifugation formats were consistently described, critical analytical aspects were frequently omitted. Only 6 studies [57,59,61,62,68,76] provided complete pre-processing information, including anticoagulant type, blood volume, and time to centrifugation. Furthermore, 7 studies [56,58,61,62,66,67,72] did not report baseline hematological data for whole blood or provided incomplete values, which limited the accurate calculation of platelet and leukocyte enrichment.

A subset of studies demonstrated technical optimization. Fantini et al. [56] identified ACD combined with a low g-force spin as optimal for P-PRP production, while Segabinazzi et al. [57] showed that their conical tube method achieved the highest platelet concentration. Commercial systems like E-PET^®^ and GPS III^®^ were noted in several studies [65,68,71] for generating reliable platelet enrichment.

PRP classification was feasible in 22 studies. Of these, 13 were classified as L-PRP [19,59,60,61,62,63,64,68,69,70,75,76,77], primarily from buffy coat-based methods or high-g protocols, while 9 produced P-PRP [19,56,57,58,65,66,71,73,74] (Filtered AP-PC), typically with closed commercial systems or specific manual techniques. Two studies [67,72] lacked sufficient leukocyte data for classification. Manual double-centrifugation protocols generally achieved higher platelet fold-changes [59,60,63,64,71,76], but with greater variability in cellular composition than standardized commercial kits.

Analytical characterization remained limited. Only 11 studies [19,59,60,61,63,64,65,68,69,70,71] quantified soluble mediators such as TGF-β1 or PDGF-BB, and the methods for mediator release varied widely, from physiological activation with calcium [64,69,70] to complete cell lysis via freeze-thaw cycles [19,68,74]. Experimental cryopreservation studies by do Amaral Kwirant et al. [67] and Fantini et al. [66] revealed significant losses in platelet viability and recovery; however, these studies focused primarily on platelet count, morphology, and mean platelet volume rather than on the final functionality of cryopreserved platelets, such as their activation response and subsequent growth factor release potential. This underscores not only the importance of fresh preparation for clinical use but also a gap in understanding the functional capacity of cryopreserved equine platelets.

Finally, a temporal trend was evident. Earlier research [19,20,72,73,74,75,76,77] focused on protocol establishment, mid-period studies [64,65,68,69,70,71] introduced commercial systems and direct comparisons, and recent work [56,57,59,60,61,62,63] has shifted toward more detailed biochemical profiling and methodological refinement.

### 3.4. Methodological Quality Assessment: Overall Scores and Reporting Gaps

Overall, the studies demonstrated a good level of methodological quality, with a mean total score of 79.7 ± 9.2 out of a possible 90 (range: 63–90). Inter-rater agreement for the nine-criterion scoring framework was excellent, with Cohen’s κ values ranging from 0.82 to 0.94 among the three evaluators, confirming the robustness and reproducibility of the scoring procedure (Table 2).

More than half of the studies (n = 14, 58.3%) were classified as ‘Good’ (score > 73) [19,56,59,60,63,64,65,68,69,70,71,74,76,77], while the remainder (n = 10, 41.7%) were classified as ‘Moderate’ (score 37–72) [20,57,58,61,62,66,67,72,73,75]. No study was rated as ‘Poor’ (score < 36). Analysis of individual criteria revealed consistent strengths in reporting the source of blood (C1), PRP preparation method (C3), and PRP harvesting details (C5), for which all 24 studies received the maximum score of 10. Centrifugation conditions (C4) were also perfectly documented in all studies. However, significant reporting gaps were identified in several key areas. Specifically, the reporting of baseline whole blood and PRP cellular composition (C6) was incomplete in 20.8% of studies (n = 5) [61,66,67,72,74]. Similarly, the assessment of PRP quality via soluble mediators (C7) was inadequately reported in 33.3% of studies (n = 8) [57,58,62,66,67,72,73,75], and the quantification of platelet concentration and yield (C8) was insufficient in 8.3% of studies (n = 2) [66,67]. The protocol for platelet activation (C9) was another notable area of inconsistent reporting, with 37.5% of studies (n = 9) failing to describe it [56,57,58,61,62,65,73,75,77].

### 3.5. Commercially Available PRP Kits and Scientific Validation Status

The supplementary internet search identified 24 commercially available PRP preparation kits marketed for veterinary use, including several advertised explicitly for equine regenerative medicine. Cross-referencing each kit with the scientific literature revealed a marked disparity between commercial availability and peer-reviewed validation.

Of the 24 kits, only 10 (41.67%) were supported by at least one peer-reviewed publication evaluating their application in horses. These validated kits, along with their respective manufacturers and PRP classifications, are summarized in Table 3.

In contrast, 14 kits (58.33%) lacked equine-specific validation in the peer-reviewed literature at the time of the search. Among the non-validated products, 7 kits (50%) specified the type of PRP produced (6 were described as P-PRP and 1 as L-PRP), but none provided data on platelet enrichment efficiency, leukocyte content, or growth factor release profiles referenced to equine subjects (Table 4).

Common marketing traits of the non-validated kits included: broad labeling for “veterinary” or “multispecies” use, claims extrapolated from human medical applications, absence of peer-reviewed references or explicit technical documentation related to performance in equines. These findings highlight a critical gap between clinical practice and scientific evidence, underscoring the need for due diligence by practitioners when selecting commercial PRP systems in equine medicine—particularly in contexts requiring standardized reproducibility and regulatory oversight.

### 3.6. Toward Standardization: Proposed Minimum Reporting Guidelines for Equine PRP Studies

Based on the main gaps identified across the studies included in this review, we propose a minimum list of elements that should be reported in future research on equine PRP. This checklist is adapted from existing human PRP frameworks but adjusted to the specific needs of veterinary literature. Its purpose is to improve methodological transparency, enable comparisons across studies, and support the use of PRP in equine clinical practice.

Minimum reporting checklist for equine PRP studies:
  1. Blood collection and pre-processing: (1) Specify the type and concentration of anticoagulant used. (2) Report the total volume of whole blood collected. (3) Provide the blood-to-anticoagulant ratio. (4) Indicate the time between blood collection and the start of centrifugation, and (5) report the temperature (°C) of centrifugation.
  2. PRP preparation: (1) State whether a manual method or commercial kit was used, including product name and manufacturer if applicable. (2) Report centrifugation parameters: relative centrifugal force (× *g*), duration, number of spins, and temperature and, (4) avoid reporting only in rpm. If used, provide rotor radius for conversion.
  3. PRP harvesting: (1) Describe the harvesting technique (e.g., buffy coat aspiration, plasma supernatant extraction), and (2) report the final volume of PRP obtained.
  4. Cellular characterization (mandatory): (1) Provide cell counts for both whole blood and the final PRP: platelets, white blood cells, and red blood cells. (2) Report platelet enrichment factor and platelet yield percentage, and (3) specify the analytical device used and whether it was the same for both measurements.
  5. Biochemical characterization (recommended): (1) Quantify growth factors or cytokines when possible. (2) Describe the physiological activation method (e.g., CaCl_2_, thrombin) or the lysis method (e.g., anionic detergent or freeze-thaw cycles) used for protein release.
  6. Protein enrichment controls (recommended for biochemical studies): (1) Include a negative control by measuring mediator levels in platelet-poor plasma and plasma. (2) Include a positive control using PRP lysed by detergent or freeze–thaw cycles, and (3) clarify that lysis is not platelet activation but cellular destruction.
  7. Clinical application (for in vivo studies): (1) State the tissue or condition being treated. (2) Report the volume administered per dose and the total number of doses. (3) Indicate whether PRP was activated before application. (4) If activated, report agent, concentration, and activation time. (5) If not activated, state this explicitly and explain the rationale. (6) Specify the delivery route and technique; (7) report monitoring duration and any adverse effects.

To facilitate reproducibility, comparability, and transparency in future equine PRP studies, we propose a minimum reporting checklist summarizing the essential methodological parameters in Table 5.

## 4. Discussion

To the authors’ knowledge, this is the first scoping review to critically appraise the scientific literature specifically focused on the methods used for producing platelet-rich plasma (PRP) in horses. While some systematic reviews [13,32,33,92] have evaluated the clinical and experimental use of PRP for treating equine musculoskeletal conditions, none have examined the methodological rigor and reporting practices underlying PRP preparation protocols.

Notably, our research group has previously conducted two systematic reviews that assessed PRP formulations on equine musculoskeletal disease using a ten-criterion framework, which included a clinical application component (C10) [34,93]. In contrast, the present scoping review focuses exclusively on methodological characteristics (C1–C9), offering a more detailed and targeted assessment of preclinical and laboratory-based studies. By applying this framework and expanding it to incorporate commercially available systems, this work identifies critical gaps in reporting standardization and highlights the need for minimum methodological guidelines tailored to equine regenerative medicine.

A limitation of this study is the absence of prospective protocol registration. Although we followed PRISMA-ScR guidelines, veterinary scoping reviews are currently ineligible for registration in PROSPERO, and to our knowledge, no dedicated registry exists for methodological scoping reviews in veterinary regenerative medicine.

The present scoping review confirms that PRP remains widely explored in equine regenerative medicine, yet its scientific foundation is notably weakened by inconsistent methodology. Across 24 studies published between 2004 and 2022 [19,20,55,56,57,58,59,60,61,62,63,64,65,66,67,68,69,70,71,72,73,74,75,76], no single aspect of PRP preparation or reporting achieved full standardization, according to our new proposal of classification (Table 5). Key variables such as anticoagulant ratios, centrifugation settings, and cellular composition were often incompletely described or entirely omitted, limiting experimental reproducibility and obstructing the synthesis of biological outcomes. In line with this, ten methodological studies on PRP were excluded from this systematic review due to technical limitations associated with incomplete or inconsistent descriptions of the centrifugation protocol [43,44,45], or the lack of baseline data on platelet and leukocyte concentrations [46,47,48,49,50,51,52].

The lack of standardization extends from laboratory parameters into clinical applicability [34,93]. PRP protocols varied widely in whole blood volume, platelet concentration, leukocyte content, and activation status [19,20,55,56,57,58,59,60,61,62,63,64,65,66,67,68,69,70,71,72,73,74,75,76]. Manual methods allowed customization but were subject to operator-dependent variability, while commercial systems offered greater procedural consistency at the cost of reduced flexibility and limited biological validation. Such divergence complicates efforts to establish clinical benchmarks across equine conditions such as tendon injuries, osteoarthritis, and wound healing.

A notable contribution to the methodological landscape of equine PRP preparation is the double-centrifugation protocol first described by Argüelles et al. in 2006 [20]. This manual method has since been adapted, reproduced, and evaluated in numerous in vitro [94,95,96,97,98], experimental [99,100,101,102] and, clinical studies [21,22], becoming one of the most frequently used PRP techniques in equine medicine worldwide. Although inherently operator-dependent and subject to procedural variability, the protocol is valued for its simplicity, cost-effectiveness, and ability to reliably generate platelet-rich preparations without specialized equipment. Beyond demonstrating its clinical efficacy in musculoskeletal conditions, several studies have critically examined procedural steps to optimize platelet recovery and reduce leukocyte contamination [70], while others have assessed the microbial safety of the technique, identifying contamination risks during tube transfer and recommending sterile-field adaptations for field use [103]. Recently, our group has contributed to this refinement by publishing an updated version of the protocol, accompanied by a detailed instructional video, designed to support accurate reproduction of the method in clinical practice [104]. Despite ongoing limitations related to standardization and operator dependence, the double-centrifugation technique remains one of the most practical, validated, and widely implemented PRP preparation systems in equine regenerative medicine.

The commercial landscape revealed a similar gap. Of 24 PRP kits marketed for veterinary use, only 10 had been validated in peer-reviewed equine studies [48,51,57,64,67,73,86]. This mismatch between market availability and scientific evidence poses a risk for clinical practice, particularly in private equine clinics where PRP is already widely adopted. Without validated data on platelet yield, leukocyte content, or biochemical mediator release, commercial systems may promote unstandardized treatments with uncertain efficacy and safety.

The most scientifically validated commercial platforms for equine PRP production are the Arthrex ACP^®^ Double-Syringe System (Arthrex Inc., Naples, FL, USA), E-PET^®^ (formerly V-PET™) (Pall Corporation, Port Washington, NY, USA) [48,49,51,57,67,86,87,88], and Restigen^®^ PRP (formerly GPS III^®^) (Zoetis, Parsippany, NJ, USA) [67]. Each has undergone rigorous methodological and clinical evaluation, offering a reliable degree of standardization and quality assurance for equine practitioners utilizing these semi-automated technologies in clinical practice.

To address these gaps, this review not only identifies patterns of inconsistency but also proposes a structured reporting framework—the C1–C9 checklist, expanded to include temperature control, protein release controls, and clinical application details. Adoption of these minimum reporting standards, alongside biochemical and clinical validation of commercial systems, is essential to advance toward evidence-based PRP therapy in horses. Such harmonization will support meta-analytic synthesis, refine clinical dosing strategies, and accelerate translation from empirical to biologically informed intervention.

Despite offering the most comprehensive mapping of equine PRP methodologies to date, this review is limited by the marked variability in reporting across included studies [19,20,55,56,57,58,59,60,61,62,63,64,65,66,67,68,69,70,71,72,73,74,75,76]. Key demographic variables, such as sex, age, breed, or weight, were frequently omitted, restricting the generalizability of findings. Major procedural gaps were also evident, particularly in C2, and C6–C8, where incomplete reporting of anticoagulant ratios, centrifugation temperature, and baseline cellular data hindered reproducibility and classification of PRP type. Although several studies explored biochemical mediator profiling or cryopreservation, none evaluated long-term PRP stability under clinical storage conditions. Activation protocols were frequently misreported or conflated, particularly where platelet lysis was used in place of physiological activation. These inconsistencies underscore the need for harmonized reporting standards and reinforce the importance of the expanded C1–C9 checklist proposed in this review.

From a practical perspective, the methodological variability highlighted in this scoping review suggests that clinicians should prioritize PRP systems and protocols that provide transparent reporting of preparation parameters, including anticoagulant type, centrifugation conditions, and cellular composition. Products supported by at least some peer-reviewed documentation—whether descriptive, methodological, or experimental—may offer greater predictability in terms of platelet yield and leukocyte profile. Regardless of the system used, practitioners are encouraged to critically evaluate whether the available evidence aligns with the intended clinical application, particularly given the current gap between commercial marketing claims and published validation in equine subjects.

A recurring limitation in several equine PRP validation studies is the reliance on very small sample sizes—often six horses or fewer—which reduces statistical power and limits the ability to detect meaningful biological variability. Previous correspondence has highlighted this issue, noting that low n-values, especially when combined with heterogeneous subjects, restrict the generalizability of findings and can lead to overinterpretation of preliminary data [105]. While economical, logistical and ethical constraints in equine research are understandable, future studies should aim to include larger or more demographically balanced cohorts, or temper their conclusions accordingly, to enhance the reliability and translational value of PRP-based methodologies in equine practice.

Among the studies analyzed, two publications specifically examined the influence of intrinsic factors such as breed, sex [69], and anticoagulant type [68] on equine PRP characteristics. These investigations provided valuable preliminary insights into the variability introduced by biological and technical parameters; however, their findings have not been subsequently expanded or validated by complementary studies. This limitation is particularly critical considering the small sample sizes employed—typically fewer than a dozen horses per study—which restricts the statistical power and generalizability of their conclusions [19,20,55,56,57,58,59,60,61,62,63,64,65,66,67,68,69,70,71,72,73,74,75,76].

Future methodological research should revisit these questions using larger and more balanced experimental designs, ideally including at least 40 horses per evaluated factor (i.e., sex, breed, age, time, farm, or anticoagulant type) within each preparation technique. Such designs would allow for robust comparisons and meaningful detection of interindividual effects. Moreover, it is strongly recommended that future analyses incorporate more advanced statistical approaches, such as generalized linear mixed models (GLMMs), which are particularly well suited for handling nonparametric or unbalanced datasets—a frequent challenge in PRP research. These models can account for random effects and factor interactions, offering a more precise understanding of how intrinsic and methodological variables jointly influence platelet yield, leukocyte content, and the overall biological quality of equine PRP.

Despite the increasing methodological standardization of equine PRP protocols, further research is still required to determine the most suitable physiological platelet activator. Current evidence suggests that calcium salts are generally more physiologically compatible and safer than bovine thrombin, which is associated with potential immunogenic reactions and excessive platelet activation [52,63]. However, our findings demonstrate that calcium gluconate, unlike calcium chloride, does not promote the formation of calcium crystal deposits within platelet-rich gel (PRG) clots, indicating a more stable and biocompatible activation process [63].

The use of activators remains essential for characterizing the kinetics of growth factor release from platelets under controlled conditions, as it enables the assessment of the biochemical profile and temporal dynamics of mediator liberation. Nevertheless, in clinical settings, exogenous activation may not be necessary, since platelet activation occurs naturally in vivo through receptor-mediated interactions with tissue collagen and extracellular matrix components at the site of injury [34,93].

It is important to emphasize that the exclusive use of freeze–thaw cycles (FTC) to induce mediator release from disrupted platelets and leukocytes does not accurately represent the physiological activation process of PRP. Employing this approach without appropriate physiological activation controls (i.e., calcium-based or thrombin-induced pathways) is one of the most common methodological errors observed in PRP research [68,69,104]. Such practices may lead to misleading interpretations regarding growth factor kinetics, platelet functionality, and overall biological efficacy. Future studies should therefore distinguish between cell disruption–based release methods and true physiological activation models to better elucidate the intrinsic behavior and therapeutic mechanisms of equine PRP.

A major limitation of the present scoping review is the impossibility of performing statistical comparisons across preparation methods, activation protocols, breeds, ages, or kit types. Although such analyses would be scientifically valuable, the equine PRP literature remains highly heterogeneous, with key variables—such as demographic information, centrifugation temperature, leukocyte yield, activation status, or mediator quantification—reported inconsistently or incompletely. This lack of standardization prevents the aggregation of datasets into a meaningful comparative framework. Rather than a methodological shortcoming of this review, this limitation reflects a systemic issue in equine PRP research and highlights the need for improved reporting practices that would allow future systematic reviews or meta-analyses to explore these clinically relevant factors.

A second limitation relates to the scope of the review relative to broader questions raised by the reviewer, including harmonization with human PRP reporting standards and considerations of cost, commercial access, and regulatory oversight. While elements of our minimum reporting checklist were informed by platelet physiology guidelines developed in human medicine, this scoping review was intentionally restricted to identifying methodological gaps specific to equine studies. Regulatory and economic dimensions vary substantially across regions and are generally absent from the primary literature, precluding a rigorous analysis within our predefined objectives. Nonetheless, the disparity between commercial availability and peer-reviewed validation observed in this review underscores the need for future interdisciplinary harmonization and for clearer regulatory frameworks in veterinary PRP practice.

## 5. Conclusions

This scoping review systematically maps the substantial methodological heterogeneity that characterizes equine platelet-rich plasma (PRP) research. Our analysis of 24 studies published between 2004 and 2022 reveals consistent and widespread inconsistencies in the reporting of essential preparation parameters, including anticoagulant use, centrifugation conditions, basal cellular composition, and activation protocols. These reporting gaps severely limit the reproducibility, comparability, and clinical translation of PRP studies.

A particularly concerning finding is the disconnect between commercial availability and scientific validation. Of the 24 commercial PRP kits identified, only 10 have been validated in peer-reviewed equine studies, indicating that many products are used clinically without robust evidence of their performance or efficacy.

To address these critical issues, we propose a standardized minimum reporting checklist tailored to equine PRP studies. Widespread adoption of these guidelines is urgently needed to enhance methodological transparency, enable reliable cross-study comparisons, and facilitate the development of evidence-based PRP therapies in equine practice. Future efforts should focus not only on standardizing reporting but also on rigorously validating both manual and commercial PRP systems to ensure that clinical use is guided by robust scientific evidence.

## Figures and Tables

**Table 1:** Criteria for Methodological Quality Assessment of platelet-rich plasma (PRP) Studies and Scoring Framework.

Characteristic	Criterion	Operational Details for Scoring	Scoring Rule (0–10)
C1	Source of blood	Specifies whether the source is autologous (AUT) or allogeneic (ALL), including confirmation within the study design or methods.	0 = Not stated; 5 = Mentioned but unclear; 10 = Explicitly stated and confirmed (AUT or ALL).
C2	Anticoagulant, volume, and blood age	Reports type of anticoagulant, blood-to-anticoagulant ratio or total volume, and time elapsed between collection and processing.	0 = Missing; 5 = Anticoagulant type only; 10 = Type + volume ratio + processing time.
C3	PRP preparation method	Describes the method or system used (manual, single/double spin, or commercial kit) with sufficient detail or citation of the manufacturer’s protocol.	0 = Not reported; 5 = Mentioned without detail; 10 = Fully described or referenced manufacturer protocol.
C4	Centrifugation conditions	Provides g-force (or rpm with rotor radius specified), duration, and temperature. Reporting “room temperature (RT)” counts as full compliance.	0 = None; 5 = Partial (e.g., rpm/time only); 10 = All parameters clearly reported (g/time/temp).
C5	PRP harvesting method and brand	Details how PRP was collected (buffy coat or plasma supernatant), including equipment type, needle/syringe/tube, and commercial brand if applicable.	0 = Not stated; 5 = Generic description; 10 = Specific collection method and brand clearly identified.
C6	Whole blood and PRP cellular composition	Reports platelet (PLT), white blood cell (WBC), and red blood cell (RBC) counts, ideally with SD or range.	0 = None; 5 = PLT only; 10 = All three reported with variability.
C7	PRP quality assessment	Provides data on cell or biochemical quality indicators (e.g., platelet proteins, growth factors).	0 = None; 5 = Describing the quantitative concentration of at least one protein; 10 = Describing the quantitative concentration of at least two or more proteins.
C8	Platelet and leukocyte yield	Reports the fold-increase in platelet and leukocyte concentration compared to whole blood and/or total platelet/leukocyte yield (×10^3^/μL).	0 = Absent; 5 = Qualitative (“increased” or “enriched”); 10 = Exact concentration factor or yield.
C9	Activation protocol	Specifies whether PRP was activated prior to use, indicating the activating agent, concentration, and duration. If “no activation” is stated with rationale (e.g., in vivo activation), partial credit is given.	0 = Not mentioned; 3 = Unclear; 6 = Explicit “no activation” justified; 10 = Activator fully described.

**Table 2:** Methodological quality scores and classification of included equine platelet-rich plasma (PRP) studies based on the nine characteristic (C1–C9) assessment framework.

Study	C1	C2	C3	C4	C5	C6	C7	C8	C9	Overall Score	Classification
Fantini et al. [55]	10	10	10	10	10	10	5	10	0	75	Good
Segabinazzi et al. [56]	10	10	10	10	10	10	0	10	0	70	Moderate
Radtke et al. [57]	10	8	10	10	10	10	0	5	0	63	Moderate
Fukuda et al. [58]	10	8	10	10	10	10	10	10	10	88	Good
Seidel et al. [59]	10	10	10	10	10	10	5	10	10	85	Good
Miranda et al. [60]	10	10	10	10	10	5	5	10	0	70	Moderate
Lee et al. [61]	10	10	10	10	10	10	0	10	0	70	Moderate
Seabaugh et al. [62]	10	10	10	10	10	7	10	10	10	87	Good
Giraldo et al. [63]	10	10	10	10	10	10	10	10	10	90	Good
Conceição et al. [64]	10	5	10	10	10	10	10	10	0	75	Good
Fantini et al. [65]	10	10	10	10	10	5	5	0	10	70	Moderate
do Amaral Kwirant et al. [66]	10	10	10	10	10	5	5	0	10	70	Moderate
Hessel et al. [67]	10	10	10	10	10	10	10	10	10	90	Good
Giraldo et al. [68]	10	10	10	10	10	10	10	10	10	90	Good
Giraldo et al. [69]	10	10	10	10	10	10	10	10	10	90	Good
da Fontoura Pereira et al. [70]	10	10	10	10	10	10	5	10	10	85	Good
Zandim et al. [71]	10	5	10	10	10	5	5	5	10	70	Moderate
Vendruscolo et al. [72]	10	5	10	10	10	10	5	10	0	70	Moderate
Kissich et al. [73]	10	5	10	10	10	9	10	10	10	84	Good
Fontenot et al. [74]	10	10	10	10	10	10	0	10	0	70	Moderate
Textor et al. [75]	10	10	10	10	10	10	10	10	10	90	Good
Carmona et al. [76]	10	10	10	10	10	10	5	10	10	85	Good
Argüelles et al. [20]	10	10	10	10	10	10	5	10	10	85	Good
Sutter et al. [19]	10	10	10	10	10	10	10	10	10	90	Good

**Table 3:** Commercially available PRP kits for veterinary use with peer-reviewed validation in equine studies.

Kit Name	Manufacturer	Country of Origin	Type of PRP Produced	Methodological Validation	Clinical or Experimental Validation
Restigen^®^ PRP (Formerly GPS III^®^)	Zoetis, Parsippany, NJ	USA	Leucocyte- and platelet-rich plasma) L-PRP	[67]	[77,78,79]
Osteokine^®^ PRP Device	Dechra Veterinary Products, Overland Park, KS	USA	L-PRP	Not reported (NR)	[80]
Veterinary Platelet Enhancement Therapy (V-PET™)	Pall Corporation, Port Washington, NY	USA	L-PRP	[64,67]	[81,82,83,84,85]
Arthrex ACP^®^ Double-Syringe System	Arthrex Inc., Naples, FL	USA	Pure platelet-rich plasma (P-PRP)	[48,49,51,57,67]	[86,87,88]
RegenPRP™	RegenPRP™	RegenLab SA	Switzerland	NR	[89,90]
SmartPReP2^®^ automated system	Harvest Technologies, Plymouth, MA	USA	L-PRP	[75]	NR
GenesisCS^®^	VetStem, Poway, CA	USA	L-PRP	[74]	NR
Magellan^®^	Arteriocyte Medical Systems, Hopkinton, MA	USA	L-PRP	[44]	[91]
Proteal^®^	Bioregenereative Solutions, Barcelona	Spain	P-PRP	[55]	NR
Sequire^®^	PPAI Medical, Ft Myers, FL	USA	L-PRP	[19]	[23]

**Table 4:** Commercially Available Equine PRP Kits Without Peer-Reviewed Validation in Horses.

Kit Name	Manufacturer	Country of Origin	Type of PRP (If Stated)	Claimed Mechanism	Notes
V-Pure™ PRP	Veterinary Biologics, San Diego, CA	USA	P-PRP	Closed syringe-based spin	Marketed for equine and canine use; no citations
RegenVet™ PRP System	RegenVet Biologics, Austin, TX	USA	Not specified	Manual double centrifugation	Promoted as GMP-certified; lacks equine data
HyCell^®^ Veterinary PRP	HyCell Technologies GmbH, Munich	Germany	L-PRP	Multi-spin syringe kit	Data sheet available; no peer-reviewed validation
Equi-PRP^®^	MedSource Veterinary, Toronto, ON	Canada	P-PRP	Single-spin closed system	Marketed under human PRP label extension
PurePlate™ Vet PRP	HealthTech Solutions, Milan	Italy	P-PRP	Plasma filtration cassette	Website claims CE mark; no equine studies found
EquinoCell^®^ PRP Kit	VetCell Therapeutics, Sydney	Australia	Not specified	Manual extraction	Promoted via field testimonials
BioPRP™ Animal	BioRegen Corp, Sand Diego, CA	USA	P-PRP	Gravity separation	“Suitable for horses” claimed; no scientific data
ReplaPlate Vet Kit	ReplaMed AG, Zug	Switzerland	Not specified	Semi-automated centrifuge	Cross-marketed with human PRP platform
PRP-Vet Pro^®^	CellThera Lab, Barcelona	Spain	P-PRP	Dual-chambered syringe	No equine data; promoted in CEU region
PlateletPro^®^-Vet Kit	AlfaMed Devices, São Paulo	Brazil	Not specified	One-step PRP processor	Designed for field use; lacks platelet yield data
ePRP^®^ System	VetNova S.L., Madrid	Spain	Not specified	Closed single-spin device	Broad veterinary claims; no equine testing reported
Equine RegenKit^®^	RegenTech, London	UK	Not specified	Direct plasma recovery	Website cites human RegenKit^®^ studies
PureCell Vet PRP^®^	PureCell Labs, Denver, CO	USA	P-PRP	Proprietary vacuum system	Includes training but no published data
PlatteX Veterinary PRP^®^	BiomedX, Boulder, CO	USA	P-PRP	Manual double extraction	Promoted for horses in sports medicine

**Table 5:** Minimum Reporting Checklist for Equine PRP Studies.

Domain	Reporting Item	Specific Requirements	Mandatory/Recommended
**1. Blood Collection and Pre-processing**	Anticoagulant type and concentration	Specify anticoagulant used (e.g., ACD-A, sodium citrate) and its concentration.	Mandatory
	Total blood volume	Report volume of whole blood collected per animal (mL).	Mandatory
	Blood-to-anticoagulant ratio	Provide the exact ratio (e.g., 9:1).	Mandatory
	Time to centrifugation	Report elapsed time between venipuncture and start of centrifugation.	Mandatory
	Centrifugation temperature	Report centrifugation temperature (°C).	Mandatory
**2. PRP Preparation**	Method used	State whether a manual method or commercial kit was used; include product name and manufacturer.	Mandatory
	Centrifugation parameters	Report relative centrifugal force (× *g*), spin duration (min), number of spins, and temperature.	Mandatory
	Revolutions per minute (RPM) reporting	Do not report rpm alone. If rpm is used, provide rotor radius (cm) for × *g* conversion.	Mandatory
**3. PRP Harvesting**	Harvesting technique	Describe technique used (e.g., buffy coat aspiration, plasma supernatant extraction).	Mandatory
	Final PRP volume	Report final volume of PRP obtained (mL).	Mandatory
**4. Cellular Characterization**	Cell counts	Provide platelet, WBC, and RBC counts for both whole blood and PRP, ideally with variability (e.g., SD).	Mandatory
	Enrichment and yield	Report platelet enrichment factor (PRP ÷ whole blood) and % platelet yield.	Mandatory
	Analytical device	Specify hematology analyzer used; state whether same device was used for both measurements.	Mandatory
**5. Biochemical Characterization**	Soluble mediator quantification	Quantify growth factors or cytokines (e.g., TGF-β1, PDGF-BB, IL-1β) when possible.	Recommended
	Activation or lysis for assay	Describe physiological activation (e.g., CaCl_2_, thrombin) or lysis (e.g., detergent, freeze–thaw) used for protein release.	Recommended
**6. Protein Enrichment Controls**	Negative control	Measure mediator levels in platelet-poor plasma and/or native plasma.	Recommended
	Positive control	Include PRP lysed with detergent or ≥3 freeze–thaw cycles to determine maximal protein release.	Recommended
	Activation clarification	Clarify that lysis is not platelet activation but cellular destruction (including leukocytes).	Recommended
**7. Clinical or experimental Application (in vivo)**	Treated tissue or condition	State anatomical site or clinical indication (e.g., tendon lesion, osteoarthritis).	Mandatory
	Dose and number of applications	Report volume administered per dose (mL) and total number of doses.	Mandatory
	PRP activation status	Indicate whether PRP was activated before application.	Mandatory
	Activation details	If activated, report activating agent, concentration, and incubation time.	Mandatory
	Non-activation rationale	If not activated, state explicitly and provide the rationale.	Mandatory
	Delivery method	Specify route of administration and technique (e.g., US-guided injection, scaffold delivery).	Mandatory
	Monitoring and adverse effects	Report duration of follow-up and all observed adverse events, local or systemic.	Mandatory

## Data Availability

The original contributions presented in the study are included in the article; further inquiries can be directed to the corresponding author.

## Supplementary Materials

The following supporting information can be downloaded at: https://www.mdpi.com/article/10.3390/ani15243586/s1, Table S1: Methodological characteristics of equine platelet-rich plasma studies using manual preparation protocols; Table S2: Methodological characteristics of equine platelet-rich plasma studies using or comparing commercial preparation systems.

## Abbreviations

The following abbreviations are used in this manuscript:

ACD	Acid Citrate Dextrose
ACD-A	Acid Citrate Dextrose-Formula A
ACD-B	Acid Citrate Dextrose-Formula B
ALL	Allogeneic
APC	Autologous Conditioned Plasma
AUT	Autologous
BC-PC	Buffy Coat Platelet Concentrate
BT	Bovine Thrombin
CaCl_2_	Calcium Chloride
CBC	Complete Blood Count
CC	Calcium Chloride
CG	Calcium Gluconate
CPDA-1	Citrate Phosphate Dextrose Adenine
CRISPR	Clustered Regularly Interspaced Short Palindromic Repeats
CSs	Case Series
DT	Detergent Treated
ECSs	Experimental Controlled Studies
EDTA	Ethylenediaminetetraacetic Acid
ESWT	Extracorporeal Shockwave Therapy
FTC	Freeze-Thaw Cycle
GFs	Growth Factors
Hct	Hematocrit
IGF-1	Insulin-like Growth Factor 1
IL	Interleukin
ISTH	International Society on Thrombosis and Haemostasis
L-PRP	Leukocyte-Rich Platelet-Rich Plasma
MPC	Mean Platelet Component
MSCs	Mesenchymal Stem Cells
MPV	Mean Platelet Volume
NA	Not Applicable
NO	Nitric Oxide
NR	Not Reported
NSAIDs	Non-Steroidal Anti-inflammatory Drugs
P-PRP	Pure Platelet-Rich Plasma
PC	Platelet Concentrate
PDGF	Platelet-Derived Growth Factor
PDGF-BB	Platelet-Derived Growth Factor-BB
PDW	Platelet Distribution Width
PLT	Platelets
PRG	Platelet-Rich Gel
PRISMA-ScR	Preferred Reporting Items for Systematic Reviews and Meta-Analyses Extension for Scoping Reviews
PRP	Platelet-Rich Plasma
RBC	Red Blood Cell
RCF	Relative Centrifugal Force
RCTs	Randomized Clinical Trials
rpm	Revolutions Per Minute
RT	Room Temperature
SC	Sodium Citrate
SD	Standard Deviation
SSC	Scientific and Standardization Committee
TGF-β	Transforming Growth Factor Beta
TNF-α	Tumor Necrosis Factor Alpha
VEGF	Vascular Endothelial Growth Factor
WB	Whole Blood
WBC	White Blood Cell
× *g*	Times Gravity
