# Supplementary material for: Addressing Heterogeneity in Equine PRP Therapies: A Scoping Review of Methods, Evidence, and Commercial Validation
Source: Animals (Basel). 2025 Dec 13;15(24):3586. doi: 10.3390/ani15243586 (PMC12729996; doi:10.3390/ani15243586)
Supplement: Supplementary file 1 [file animals-15-03586-s001.zip › Table S1.pdf]

Table S1. Methodological characteristics of equine platelet-rich plasma studies using manual preparation protocols.

Study & year	C1	C2	C3	C4	C5	C6	C7	C8	C9	Observations
Segabinazzi et al. [56], 2021	AUT	150 mL of whole blood (WB) collected in a transfusion bag with citrate-phosphate-dextrose solution with adenine (CPD-A), 32.4 mL of WB collected into 3.2% SC tubes, and 60 mL of WB collected in a syringe with CPD-A. Immediate platelet-rich plasma (PRP) preparation	Manual methods (M) (M1 (conical tubes of 50 mL (first spin), and 15 mL (second spin), M2 (2.7 mL SC tubes), M3 (syringe sedimentation)	M1: 400 × g/15 min and 1000 × g/10 min; M2: 120 × g/10′; M3: sedimentation over 4 h	Manual buffy coat harvesting	WB, platelets (PLT)/μL: 125.8 ± 37.6 × 10³, white blood cells (WBC)/μL: 5.4 ± 1.4 × 10³. PRP, M1, PLT/μL: 709.4 ± 167.3 × 10³, M2, 327.5 ± 67.9 × 10³, M3, 239.4 ± 45.6 × 10³; M1, WBC/μL: 0.027 ± 0.015 × 10³, M2, 0.050 ± 0.026 × 10³, M3, 1.900 ± 1.220 × 10³	No cytokines or growth factors (GFs) evaluated; pH and biochemical stability of PRP after 24h at 4°C assessed	PLT yield: 5.64 × (M1), 2.52 × (M2), 1.85 × (M3); WBC yield: 0.005 × (M1), 0.009 × (M2), 0.35 × (M3)	Not applicable (NA). This study did not include mediator measurement in PRP releasates	This study compared three manual PRP preparation protocols (M1–M3) in horses. Method 1 (140g/10′) showed the highest platelet concentration and lowest leukocyte contamination. PRP quality was monitored after 24 h refrigeration, with no significant changes in pH, platelet count, or plasma protein. No growth factors or cytokines were analyzed. The study supports M1 as optimal for pure platelet-rich plasma (P-PRP) preparation under field conditions. n: 18. Classification: P-PRP
Fukuda et al. [58], 2020	AUT	100 mL of WB collected into syringes containing 10% acid-citrate-dextrose solution (ACD-A)	Manual double-spin method	1st spin: 400 × g/7 min at 4°C. 2nd spin: 2,000 × g/7 min at 4°C	Manual harvesting. Plasma fraction was transferred after the first spin. The pellet was	WB: PLT: 120 ± 20 x 10³/μL; WBC: 6.7 ± 1.2 x 10³/μL. PRP, PLT: 920 ± 160 x 10³/μL; WBC: 3.7 ± 1.6 x 103/μL	Platelet-derived growth factor BB (PDGF-BB): Significantly higher in calcium (Ca)-activated PRP (5,222 ± 3,957 pg/mL) vs. Frozen (Fr) (1,369 ± 1,138 pg/mL). Transforming growth factor beta 1 (TGF-β_1): Significantly higher in Fr	PLT yield: 7.4x ± 0.9. WBC yield: 0.6x	M1, 10% calcium chloride ($CaCl_2$) + autologous serum (1:3 ratio) to PRP (1:10), incubated at 37°C for 5 min.	Study focused on optimizing PRP activation to maximize GF release. Key findings: The Ca method yielded the highest PDGF-BB concentration. TGF-β_1 release was high with both Ca and, FTC (double freeze-thawing) was optimal for cryopreserved PRP,

					resuspended in 1 mL of supernatant after the second spin. No commercial device was used		(7,235 ± 2,842 pg/mL) and Ca (8,084 ± 2,257 pg/mL) vs. non-activated PRP		M2, Freeze-thaw cycle (FTC).	significantly increasing both GFs compared to a single cycle. Growth factors remained stable after 1 month of cryopreservation. n: 11. Classification: leukocyte- and platelet-rich plasma (L-PRP)
Seidel et al. [59] 2019	AUT	WB collected into tubes with 3.8% sodium citrate (9:1 ratio). Volumes: 10 mL (single protocol) or ~31.5 mL (double protocol). Processed fresh	Manual protocols: M1, single centrifugation. M2, double centrifugation with sequential spins and rest periods	Single: 140 × g for 12 min of resting time (RT). Double: 1st spin 300 × g for 5 min (RT 25 min), 2nd spin 700 × g for 15 min (RT 45 min), plus additional clarification (1000 × g/10 min)	Manual pipette transfers, Falcon tubes. No commercial brand used	WB, PLT baseline: ~180 $\times 10^3/\mu L$. WBC: 7.5 $\times 10^3/\mu L$ PRP-0 (single): 494 ± 157 $\times 10^3/\mu L$ (2.7×). Platelet-poor plasma (PPP)-1: 756 ± 228 $\times 10^3/\mu L$. WBC: 0.2 × $10^3/\mu L$. PRP-1: 1371 ± 423 $\times 10^3/\mu L$. WBC: 4.2 ± 1.8 × $10^3/\mu L$	TGF-β_1, PRP-0: 5537 pg/mL; PPP-1: 5539 pg/mL; PRP-1: 12,407 pg/mL; PPP-2: 3102 pg/mL. Significant positive correlation between platelet counts and TGF-β_1 (R^2 = 0.74, r = 0.86)	PLT enrichment: 2.7× (PRP-0), 4.2× (PPP-1), 7.6× (PRP-1). Leukocytes not enriched; counts reduced compared to WB, but detectable (PRP1: ~4.5 $\times 10^3/\mu L$; WBC yield ~0.5)	Aggregometry with collagen type I (exogenous agonist). PRP-0: high aggregation (110%); PRP-1: reduced aggregation (66.8%), indicating premature activation during double centrifugation	This study compared single and double centrifugation for equine PRP. Double centrifugation achieved the highest platelet and TGF-β_1 levels but showed premature activation, while single centrifugation produced lower values with less activation. All products were leukocyte-poor. The study was limited by a small sample size (n=12), focus on TGF-β_1 only, and absence of in vivo validation, but it highlighted the strong effect of centrifugation intensity on PRP quality. Classification, PRP0: P-PPRP, PRP1: L-PRP.
Miranda et al. [60], 2019	AUT	60 mL WB, collected in tubes with 3.2% sodium citrate; processed within 2 h	Manual double-spin method; two protocols differing in resting period	M1, 120 g/10 min. M2, 240 g/10 min. Rest period applied in different stages	Buffy coat aspirated manually using pipettes. No commercial device brand reported	WB, PLT: 244.2 ± 21.3 $\times 10^3/\mu L$ (manual), 256.5 ± 37.8 $\times 10^3/\mu L$ (automated). WBC: Not reported (NR). M1, PRP: 463.0 ± 71.0 $\times 10^3/\mu L$, WBC: increased by 345.3%, and red	Vascular-endothelial growth factor (VEGF): M1: 0.82 ± 0.04 ng/mL; M2: 0.84 ± 0.04 ng/mL	M1: 2.02× enrichment. M2 3.18× enrichment. WBCs: Increased in both protocols	NR	This trial showed that protocol design, particularly a pre-centrifugation resting period, significantly improves PRP yield while maintaining VEGF stability. Limitations include small sample size, restriction to Quarter Horses, and assessment of only one growth factor. Manual

						blood cells (RBC) decreased by 8.1%. M2, PLT: 761.8 ± 41.9 ×10^3/μL, WBC: increased by 525.6%, and RBC: decreased by 8.1%.				handling and absence of activation reduce comparability with other protocols. n: 8. Classification: L-PRP for both protocols
Lee et al. [61], 2018	AUT	WB collected into syringes containing ACD-A anticoagulant. Volume not explicitly stated for each preparation. Processed immediately after collection	Compared 3 manual methods. M1, two-step centrifugation, M2, separated centrifugation, and M3, separated centrifugation using Histopaque® for leukocyte isolation	Step 1: 200 × g/5 min; Step 2: 900 × g/15 min. For the Histopaque® group, an additional 400 × g/30 min, then 200 × g/10 min wash. All at room temperature	Manual transfer using pipettes and Falcon tubes; Histopaque® used for gradient separation.	WB, PLT: 101.8 ± 23.4 × 10^3/μL, WBC: 7.59 ± 1.00 × 10^3/μL, RBC: 7.76 ± 1.75 × 10^6/μL. PRP, M1: PLT: 456.3 ± 116.2 × 10^3/μL, 10^3/μL, RBC: 8.50 ± 2.31 × 10^6/μL. M2: PLT: 542.5 ± 97.7 × 10^3/μL, WBC: 18.1 ± 4.0 × 10^3/μL, RBC: 3.39 ± 1.19 × 10^6/μL. M3, PLT: 569.2 ± 108.6 × 10^3/μL, WBC: 12.5 ± 2.6 × 10^3/μL, RBC: 0.09 ± 0.05 × 10^6/μL	No cytokines or GFs evaluated	Platelets concentrated up to 5.6× baseline. WBC: highest in 2-step centrifugation (35.2 ± 3.6 ×10^3/μL), intermediate in separated centrifugation (18.1 ± 4.0 ×10^3/μL), lowest in Histopaque (12.5 ± 2.6 ×10^3/μL). RBCs lowest in Histopaque (0.09 ± 0.05 ×10^6/μL)	NA. This study did not include mediator measurement in PRP releasates	This study compared three manual PRP methods, emphasizing cellular composition rather than growth factor content. The Histopaque®-based method achieved the lowest RBC and WBC concentrations. The two-step centrifugation produced higher WBC and RBC levels, yielding a more leukocyte-rich product. The study was limited by a small sample size (n=6), lack of biochemical assays, and reliance solely on hematologic parameters without activation or functional testing. Classification: L-PRP for all methods
Seabaugh et al. [62], 2017	AUT	WB collected into 450 mL CPDA-1 blood donor bags and processed within hours of collection. An additional 7	Manual sequential centrifugation (3-step) performed under sterile laminar flow	1st spin = 700 g × 10 min (4 °C, no brake); 2nd = 1250 g × 5 min (4 °C); 3rd = 2370 g × 5 min (4 °C). PPP	Manual aspiration and combination of platelet pellets; resuspension with PPP. No	WB, PLT: 180 ± 30 ×10^3/μL, WBC: NR, RBC: NR. PRP–final preparation after sequential centrifugation: PLT: 1,037 ± 300 ×10^3/μL, WBC: 0.84 ± 0.5 ×10^3/μL, RBC: 0.06 ± 0.02 ×10^6/μL	TGF-β_1 – Positive control 7063 (5040–10 042); ESWT-S 2101 (1488–3357); ESWT-P 2000 (1669–2785); Negative 1485 (1012–2501). PDGF-BB – Positive 4107 (3718–4229); ESWT-S 566 (408–724); ESWT-P 405 (331–	PRP enrichment ≈ 4–5× above baseline; leukocytes and RBCs both minimal. Target platelet yield standardized to 1 × 10^6/μL by dilution	Compared 4 activation conditions: (1) freeze–thaw (positive control), (2) resting PRP (negative control), (3) extracorporeal	This in vitro study tested whether ESWT increases growth factor release from equine PRP. Both probes elevated TGF-β_1 and PDGF-BB compared with controls, though less than freeze–thaw activation. The PRP was leukocyte-poor with standardized platelet counts

		mL EDTA sample used for cell blood count (CBC)	conditions. Aimed to produce PRP at 1000 × 10^3 PLT/μL	separated after final spin and PRP diluted to target concentration	commercial PRP kit; all procedures performed in-house with sterile conical tubes		593); Negative 174 (94–307)		shock-wave therapy (ESWT)-S (standard probe): 0.12 mJ/mm²), and (4) ESWT-P (power probe: 0.28 mJ/mm²). Each received 300 pulses at 23 kV / 2 Hz in a water-bath chamber	(~1 × 10^6/μL). Despite a small sample size (n=6) and in vitro limitations, findings indicate ESWT may serve as a practical, non-chemical activator for combined PRP–ESWT therapy in equine tendon and ligament repair. Classification: L-PRP
Giraldo et al. [63], 2017	AUT	WB (~330 mL per horse) collected into ACD-A (8.5 mL Vacutainer® tubes). Processed immediately	Manual 2-step centrifugation (soft-spin protocol)	Step 1: 120 × g/5 min and; step 2: 240 × g/5 min	Manual collection in silicone-coated glass tubes (BD Vacutainer®).	WB: PLT 141.8 ± 23.2 ×10^3/μL; WBC 7.0 ± 0.8 ×10^3/μL. PRP: PLT 363.1 ± 66.9 ×10^3/μL; WBC 3.0 ± 1.8 ×10^3/μL	GF concentration from platelet-rich gel releasates (PRGs). $CaCl_2$, ~PDGF-BB: 15,000 pg/mL, ~TGF-β_1: 7,000 pg/mL. Calcium Gluconate (CG), ~PDGF-BB: 10,000 pg/mL, ~TGF-β_1: 8,000 pg/mL. Bovine Thrombin (BT): ~PDGF-BB: 13,000 pg/mL, ~TGF-β_1 7,500 pg/mL.BT+ $CaCl_2$, ~PDGF-BB: 14,000 pg/mL, ~TGF-β_1: 6,000 pg/mL. BT+CG, ~PDGF-BB: 12,000 pg/mL, ~TGF-β1: 8,000 pg/mL	Platelet enrichment ≈ 2.6× relative to WB; leukocytes reduced >50%	Compared 5 activators: $CaCl_2$, CG, BT, and their combinations Activation ratio 10:1 (PRP: activator). Incubated at 37°C for 24 h; supernatants collected at 6, 12, and 24 h	This study compared five PRP activators to assess PDGF-BB and TGF-β_1 release. All agents increased growth factors versus plasma; CG gave the most stable clots without calcium deposits, while $CaCl_2$ caused precipitation and thrombin led to rapid retraction. Though based on more animals than prior work. Overall, CG was identified as the most effective and stable PRP activator. n: 20. Classification: L-PRP
Fantini et al. [65] 2016	AUT	4.5 mL of WB collected into sodium citrate (SC) tubes; processed immediately after collection	Manual single centrifugation protocol	133 × g for 8 min; deceleration (brake) for 2 min; temperature not reported	Plasma aspirated 4 mm above buffy coat, pooled into microtubes via 14G syringe	WB, PLT: ~165 × 10^3/μL; PRP: ~463 × 10^3/μL (~2.8×). WBC increased by ~345% in PRP (absolute counts not reported). RBC: NR	Morphology showed increased platelet activation and pseudopodia after freezing without dimethyl sulfoxide (DMSO); DMSO preserved discoid shape. Mean platelet volume	PLT enrichment: ~2.8× WB (fresh). Recovery after thawing decreased to ~70% (DMSO) and ~50% (no DMSO) after 90 days	FTC	This study demonstrated that cryopreservation alters PRP morphology and platelet recovery, especially without DMSO. Storage at −20 °C was less effective than −80 °C. Classification: L-PRP

					and catheter; no cryopreservation performed		(MPV) and PLT aggregation decreased with storage duration			
do Amaral Kwirant et al. [66], 2016	AUT	500 mL of WB collected into CPDA anticoagulant bags; processed immediately after collection	Manual double centrifugation	1st spin = 224 × g/10 min; 2nd spin = 440 × g/10 min. Both at room temperature	Manual pipetting into sterile 50 mL polypropylene tubes; cryopreservation in 2 mL Eppendorf tubes with or without 6% DMSO	WB, PLT: 167.8 ± 23.3 $\times 10^3$/µL. Fresh PRP: 830 ± 95.3 $\times 10^3$/µL (≈5.2×). 6% DMSO PRP: 617.9 ± 65.5 $\times 10^3$/µL (≈4.0×). Frozen PRP (no DMSO): 519.6 ± 66.1 $\times 10^3$/µL (≈3.2×). WBC counts not reported	MPV: WB = 6.0 ± 0.1 fL; Fresh PRP = 5.2 ± 0.07 fL; DMSO PRP = 5.3 ± 0.06 fL; Frozen PRP = 5.7 ± 0.08 fL. Morphology: fresh PRP 4% activated; DMSO PRP 9.5%; frozen PRP 13.9%. DMSO preserved discoid shape; freezing alone increased pseudopodia formation	FTC	Platelet recovery after thawing: 74.5% (DMSO PRP) vs 62.6% (no DMSO). Both PRP types contained ≥3× baseline platelet concentration; leukocytes not assessed	This study evaluated the use of 6% DMSO as a cryoprotectant for equine PRP stored at –80°C for 14 days. The double centrifugation protocol produced PRP with 3–5× platelet enrichment. Samples frozen with DMSO maintained platelet count, morphology, and MPV similar to fresh PRP, while those frozen without DMSO showed decreased platelet recovery, higher MPV, and greater activation. n=8. Classification: (WBC PRP concentrations not reported)
Giraldo et al. [68], 2015	AUT	WB collected in triplicate (110–140 mL total) deposited in Vacutainer® tubes containing either SC, ACD-A, or acid-citrate dextrose solution B (ACD-B). Processed	Manual double centrifugation tube method	Step 1: 120 × g/5 min (first 50% plasma fraction collected); Step 2: 240 × g/5 min (bottom quarter collected as PRP).	Manual collection and transfer between BD Vacutainer® tubes (SC, ACD-A, or ACD-B). All handled under sterile conditions	WB: PLT 138–145 $\times 10^3$/µL; WBC 7.9–8.4 $\times 10^3$/µL. PRP: PLT 390–400 $\times 10^3$/µL (~2.7–2.8× enrichment); WBC similar to WB (9–10 $\times 10^3$/µL). PPP: PLT ~110 $\times 10^3$/µL. Anticoagulant type did not significantly affect cell counts	SC: PDGF-BB values were 0.9 ± 0.6 in plasma, 25.2 ± 14.4 in PRP lysate, 19.0 ± 29.4 in PRGs, 8.3 ± 5.4 in PPP lysate, and 4.5 ± 5.5 in platelet-poor gel supernatant (PPGs), while TGF-β_1 reached 26.8 ± 10.4, 90.7 ± 30.7, 54.5 ± 33.1, 45.2 ± 10.3, and 29.2 ± 17.1 respectively. ACD-A: PDGF-BB concentrations were 1.0 ± 0.6, 28.2 ± 20.1, 11.3 ± 30.6, 8.2 ± 5.0, and 5.8 ± 8.0, with TGF-β_1	Platelet enrichment ≈ 2.7×; WBC counts unchanged versus WB. ACD-B produced slightly lower PLT and GF values	CG (10%) used as activator (400 µL per 4 mL PRP). Lysates prepared with 0.5% Triton X-100 as positive control	This study evaluated the effects of three anticoagulants (SC, ACD-A, and -B) on cell counts and growth factor release from equine P-PRP and PRG. Anticoagulant type did not significantly influence platelet or leukocyte counts, nor PDGF-BB or TGF-β_1 concentrations. ACD-B produced slightly lower cellular and molecular yields. CG activation efficiently induced growth factor release, while platelet

		within 1 hour			and transported on ice		values of 30.0 ± 8.7, 101.5 ± 31.2, 56.4 ± 39.1, 47.8 ± 12.2, and 33.3 ± 17.9. ACD-B: PDGF-BB values were 1.1 ± 0.9, 18.4 ± 13.4, 6.6 ± 17.3, 7.2 ± 4.1, and 4.8 ± 6.5, and TGF-β_1 reached 27.5 ± 10.0, 87.8 ± 23.0, 50.8 ± 31.2, 44.6 ± 12.9, and 31.3 ± 12.7 for the same respective components			lysates yielded the highest concentrations overall. n: 18. Classification: L-PRP.
Giraldo et al. [69], 2013	AUT	WB collected into 8.5 mL BD Vacutainer® tubes containing ACD-Aas anticoagulant. Samples were processed immediately after collection	Manual double centrifugation tube method	1st centrifugation: 120 × g/5 min (collection of the top 50% plasma adjacent to the buffy coat). 2nd centrifugation: 240 × g/5 min (bottom 25% fraction collected as PRP).	Manual transfer of plasma fractions performed with sterile syringes.	WB: PLT 167.1 ± 25.9 $\times10^3$/μL; WBC 8.2 ± 1.6 $\times10^3$/μL. PRP: PLT 304.3 ± 43.9 $\times10^3$/μL; WBC 4.3 ± 2.2 $\times10^3$/μL. Mean platelet volume (MPV) 3.6 ± 1.0 fL; platelet distribution width (PDW) 17.1 ± 0.5%.	TGF-β_1 concentrations (pg/mL): plasma 1114 ± 458, PRP lysate 3193 ± 1395, PRP: 2556 ± 1069, PDGF-BB (pg/mL): PRP lysate 1260 ± 419, PRP 1229 ± 41	PRP showed 1.8× platelet enrichment and 50% WBC reduction compared to whole blood, confirming a leukocyte-poor profile. Platelet yield and growth factor content were influenced by breed, sex, and age. Young horses and Colombian Creole females exhibited significantly higher PDGF-BB levels ($P < 0.001$)	Calcium gluconate 10% (600 μL per 6 mL P-PRP or PPP) used as activator. Samples incubated at 37°C for 6 h, then centrifuged to recover supernatants (P-PRG and PPG). Positive control lysates generated with 0.5% Triton X-100 (NID) for total growth factor release	This study analyzed the influence of breed, sex, and age on equine P-PRP and P-PRG prepared by manual double centrifugation. The protocol produced moderate platelet enrichment (1.8×) and reduced leukocytes by about 50%, yielding a leukocyte-poor PRP. CG activation induced near-complete release of TGF-β_1 and PDGF-BB within six hours. Younger and female Colombian Creole horses showed higher PDGF-BB concentrations, suggesting intrinsic variability in platelet growth factor content. Both growth factors correlated with platelet counts, confirming platelets as the primary source. n: 40. Classification: L-PRP
da Fontoura Pereira et al. [70], 2013	AUT	WB collected using two 450 mL CPDA-1	Manual double-centrifugation	PI: 120 × g / 5 min and 240 × g / 5 min; PII:	Manual plasma transfer performed	WB, PLT: 150–160 $\times10^3$/μL. PRP PLT counts averaged 4.0–5.4× baseline	TGF- β_1 (pg/mL): PI 14,053 ± 5862; PVII 12,397 ± 1517; PVI 10,518 ± 2515; PV 10,004 ± 2440; PIV	All protocols achieved platelet enrichment of 4–5× WB, meeting the	FTC	This study compared seven manual double-centrifugation protocols for equine PRP preparation to

		blood bags (citrate/phosphate/dextrose/adenine) per horse. Samples processed immediately after collection	technique. Seven different protocols (PI–PVII) were evaluated, each varying in relative centrifugal force (g) and spin duration. All produced approximately 10 mL of PRP (≈10% of original volume)	120 × g / 10 min and 240 × g / 10 min; PIII: 300 × g / 10 min and 640 × g / 10 min; PIV: 400 × g / 10 min and 800 × g / 10 min; PV: 224 × g / 10 min and 440 × g / 10 min; PVI: 113 × g / 10 min and 652 × g / 5 min; PVII: 120 × g / 5 min and 473 × g / 5 min	in Falcon 50 mL polypropylene tubes under laminar flow. PRP collected and stored in Eppendorf tubes at −80 °C for TGF-β1 quantification	across protocols: PVI 5.4×, PIV 4.8×, PV 4.8×, PII 4.7×, PIII 4.6×, PVII 4.5×, PI 4.1×. Mean PRP platelet concentration: 618,757 ± 91,630/μL. Leucocyte contamination was highest in PI and lowest in PIII and PIV; RBC contamination followed the same trend	8796 ± 2294; PII 7634 ± 1218; PIII 7198 ± 2996	therapeutic threshold. Protocol I showed the highest leukocyte and RBC contamination, while PIII and PIV yielded cleanest PRP samples. PVI produced the greatest platelet yield but with moderate WBC content		determine their efficiency in platelet concentration, leukocyte contamination, and TGF-β_1 yield. All protocols produced similar platelet enrichment (4–5× baseline), confirming their suitability for therapeutic PRP preparation. TGF-β_1 concentrations did not correlate with platelet counts, suggesting variable platelet degranulation efficiency. Protocol I was inadequate due to excessive RBC and WBC contamination, while PIV, PV, and PVI achieved higher platelet counts and cleaner plasma fractions. Despite slight differences in TGF-β1 among protocols, all met expected concentrations for biological use. The study concluded that manual protocols with moderate g-forces and longer centrifugation times are optimal for equine PRP, with PIV, PV, and PVI offering the best balance between platelet recovery and purity. n: 10. Classification: PIV: P-PRP, PV: P-PRP, PVI: L-PRP
Zandim et al. [71], 2012	AUT	Blood collected into 18 vacutainer tubes (4.5 mL blood +	Manual double centrifugation	1st centrifugation: 120 × g/5 min; 2nd centrifugat	PRP fractions manually transferred under	WB, PLT: 145–188 ×10³/μL. PRP, PLT: 310–650 ×10³/μL (≈2–4× enrichment). Erythrocytes and	Platelet morphology evaluated by transmission electron microscopy (TEM). Pure PRP: 41% resting, 49% uncertain activation, 9%	PRP platelet counts 2–4× WB. Leucocyte and RBC contamination variable; presence of polymorphonuclear	Three pharmacologic activators tested: 10% $CaCl_2$, bovine thrombin (20	This ultrastructural study compared platelet morphology in equine PRP activated with $CaCl_2$, bovine thrombin, both combined, or left inactivated. $CaCl_2$

		0.5 mL sodium citrate 3.8%, 0.199 mol/L) per horse		ion: 240 × g/ 5 min.	sterile conditions	polymorphonuclear leukocytes present in all samples; mononuclear leukocytes observed only in bovine thrombin-treated PRP	fully activated, 1% irreversibly damaged. $CaCl_2$ (10%) PRP: 20% resting, 54% uncertain, 24% fully activated, 2% irreversibly damaged. Bovine thrombin and $CaCl_2$ + thrombin caused irregular shapes, ruptured membranes, and extracellular granules not fitting the standard classification. Treatment significantly affected platelet morphology ($P = 0.03$)	cells noted. Aggregates occasionally observed in pure PRP and $CaCl_2$ + thrombin samples	IU), and their combination; compared with inactivated PRP. All incubated at 20–22°C for 2 h to promote degranulation	yielded the highest proportion of fully activated platelets (24%) without excessive damage, confirming it as an effective activator. Bovine thrombin and $CaCl_2$ + thrombin caused platelet rupture, irregular forms, and extracellular granules, suggesting overactivation. Pure PRP contained many resting and semi-activated platelets capable of activation by endogenous stimuli, making it suitable for liquid applications. Results support $CaCl_2$ as the most physiologic activator, while inactivated PRP remains viable for in situ activation during therapy. n: 4. Classification: None (WBC PRP concentrations not reported)
Vendruscolo et al. [72] 2012	AUT	27 mL WB in 3.2% sodium citrate; processed within 5 min	Manual double centrifugation	Ten Ps, 1: 120 g / 5 min then 240 g / 5 min. 2: 120 g / 10 min then 240 g / 10 min. 3: 90 g / 5 min then 180 g / 5 min. 4: 90 g / 10 min then 180 g / 10 min. 5:	Top 50% discarded as PPP; bottom 50% collected as PRP; pipetted manually into Eppendorf tubes	WB, PLT: 156.6 $\times 10^3/\mu L$; WBC: 8.3 $\times 10^3/\mu L$. P1, PLT: 205 $\times 10^3/\mu L$; WBC: 4.90 $\times 10^3/\mu L$. P2, PLT: 210 $\times 10^3/\mu L$; WBC: 2.46 $\times 10^3/\mu L$. P3, PLT: 245 $\times 10^3/\mu L$; WBC: 12.96 $\times 10^3/\mu L$. P4, PLT: 229 $\times 10^3/\mu L$; WBC: 4.15 $\times 10^3/\mu L$. P5, PLT: 280 $\times 10^3/\mu L$; WBC: 2.49 $\times 10^3/\mu L$. P6, PLT:	P1, TGF-β: 453.10 ± 43.18 pg/mL; P2, TGF-β: 506.23 ± 198.57 pg/mL; P3, TGF-β: 839.23 ± 206.91 pg/mL; P4, TGF-β: 541.15 ± 143.97 pg/mL; P5–P10, TGF-β: NT	P1, PLT yield: 1.31×; WBC yield: 0.59×; P2, PLT yield: 1.34×; WBC yield: 0.30×; P3, PLT yield: 1.56×; WBC yield: 1.56×; P4, PLT yield: 1.46×; WBC yield: 0.50×; P5, PLT yield: 1.79×; WBC yield: 0.30×; P6, PLT yield: 1.88×; WBC yield: 0.12×; P7, PLT yield: 2.11×; WBC yield: 0.13×; P8, PLT yield: 2.17×;	NR	This study compared ten manual double-centrifugation protocols for equine PRP preparation and found that the low g-force protocols (P1–P4) provided the most effective balance between platelet concentration (≈2.0–2.3× baseline) and controlled leukocyte levels, while also yielding higher TGF-β concentrations (≈450–840 pg/mL). Higher g-force protocols (P7–P10) resulted

				120 g / 5 min then 473 g / 5 min. 6: 120 g / 10 min then 473 g / 10 min. 7: 300 g / 5 min then 640 g / 5 min. 8: 300 g / 10 min then 640 g / 10 min. 9: 350 g / 5 min then 510 g / 5 min. 10: 350 g / 10 min then 510 g / 10 min		295 $\times 10^3$/μL; WBC: 0.96 $\times 10^3$/μL. P7, PLT: 330 $\times 10^3$/μL; WBC: 1.08 $\times 10^3$/μL. P8, PLT: 340 $\times 10^3$/μL; WBC: 0.39 $\times 10^3$/μL. P9, PLT: 350 $\times 10^3$/μL; WBC: 2.29 $\times 10^3$/μL. P10, PLT: 364 $\times 10^3$/μL; WBC: 0.45 $\times 10^3$/μL		WBC yield: 0.05×; P9, PLT yield: 2.23×; WBC yield: 0.28×; P10, PLT yield: 2.32×; WBC yield: 0.05×.		in excessive leukocyte reduction and lower platelet recovery, making them less suitable for consistent PRP production. Growth factor data were only reported for P1–P4, showing no statistical differences among them. No activation step or erythrocyte quantification was performed. n: 5. Classification: L-PRP (P1-5 and 9); P-PRP (P6, 7 and 10)
Carmona et al. [76], 2008	AUT	WB (125 mL per horse) collected using 25 vacuum tubes with 3.8% SC (0.5 mL citrate + 4.5 mL blood per tube). Samples processed within 30 minutes of collection	Manual single- and double-centrifugation	Single centrifugation: 120 × g for 5 min (producing platelet concentrate (PC)-A and PC-B). Double centrifugation: first 120 × g for 5 min, second 240 × g for 5 min (producin	Plasma fractions manually aspirated using 18G spinal needles attached to 20 mL syringes; transferred to 10 mL polypropylene tubes	WB: PLT 156 (91–276) $\times 10^3$/μL; WBC 7.0 (5.1–11.0) $\times 10^3$/μL. PLT counts differed significantly among all PCs. PC-A 230 (127–365), PC-B 229 (124–359), PC-C 273 (139–480), PC-D 190 (111–294) $\times 10^3$/μL ($P < 0.05$). Leukocytes: PC-A 4.0 (3.5–5.0), PC-B 3.0 (1.7–5.1), PC-C 8.5 (3.5–18.3), PC-	Only 18.4% of samples had detectable TGF-β_3 levels (>30 pg/mL); the remaining 81.6% were below detection threshold. Mean detectable range <30–1,169 pg/mL; no statistical differences among PCs or plasma. Nitric oxide (NO) values (μM): WB 33.8 ± 9, PC-A 37.1 ± 12.5, PC-B 34 ± 10, PC-C 35 ± 8, PC-D 35 ± 11, with no significant differences	PLT counts 1.2–1.8× WB, confirming mild enrichment. PC-C yielded highest PLT and WBC concentrations. Leukocyte profiles varied widely between fractions, consistent with manual layer extraction. No correlation between platelet or leukocyte numbers and TGF-β_3 or NO concentrations	Chemical activation with 10% $CaCl_2$ (250 μL per 5 mL PRP) for 2 h at 20–22°C	This study evaluated single- and double-centrifugation tube methods for concentrating platelets, leukocytes, TGF-β_3, and nitric oxide in equine blood. All platelet concentrates showed significant increases in platelet and leukocyte counts compared with whole blood, confirming the reproducibility of both protocols. However, TGF-β_3 concentrations were mostly below the ELISA detection limit (<30 pg/mL) and showed no correlation with platelet or leukocyte counts,

				g PC-C and PC-D). Post-activation spin: 1,720 × g for 8 min to separate plasma supernatant from fibrin clot		D 0.9 (0.6–2.5) ×10^3/μL				indicating that this isoform is scarce in equine PRP. Nitric oxide levels were consistent across all fractions and similar to plasma, suggesting that the centrifugation process did not trigger leukocyte activation or oxidative stress. n: 26. Classification: L-PRP
Argüelles et al. [20], 2006	AUT	125 mL of WB collected into 25 tubes containing 3.8% SC (4.5 mL blood + 0.5 mL citrate). Samples processed within 30 min after collection	Manual single and double centrifugation tube methods	Single centrifugation: 120 × g/5 min. PC-A: lower 50%, PC-B: upper 50%). Double centrifugation: first 120 × g for 5 min, second 240 × g for 5 min, PC-C: lower 25%, PC-D: upper 75%)	Manual aspiration with 18G spinal needle and 20 mL syringe; plasma transferred to polypropylene tubes.	WB, 158 × 10^3 PLT/μL, 7.1 × 10^3 WBC/μL, packed cell volume (PCV) of 26.3%. The single-spin PCs showed 229 × 10^3/μL PLT, 4.1 × 10^3/μL WBC, 0.0% PCV in PC-A. PC-B: 228 × 10^3/μL PLT, 3.1 × 10^3/μL WBC, 0.1% PCV. PC-C with 272 × 10^3/μL PLT, 8.4 × 10^3/μL WBC, 0.2% PCV. PC-D with 191 × 10^3/μL PLT, 0.93 × 10^3/μL WBC, 0.0% PCV.	TGF-β_1 (ng/mL): WB 8.3 (1.8–16.4); PC-A 9.4 (1.5–17.8); PC-B 10.3 (1.8–17.9); PC-C 10.5 (1.9–17.1); PC-D 9.9 (1.2–16.2). Platelet parameters: MPV 13.0 ± 1.0 fL, mean platelet component (MPC) 16.5 ± 1.5 g/dL, unchanged platelet distribution width (PDW) ≈57%	Platelet enrichment ranged 1.2–1.7× WB. PC-C had the highest platelet and leukocyte counts. Platelet collection efficiency: PC-A 68%, PC-B 67%, PC-C 10%, PC-D 47%. TGF-β1 efficiency: PC-A 54%, PC-B 58%, PC-C 7.5%, PC-D 43%	Calcium chloride 10% (250 μL/5 mL PRP) for 2 h at room temperature	This study evaluated single- and double-centrifugation tube methods for preparing PCs. Both techniques significantly increased platelet counts over WB, though enrichment was modest (≤1.7×). PC-C achieved the highest platelet and leukocyte recovery, while PC-D was the cleanest fraction. TGF-β_1 concentrations rose 38–44% compared to WB but were unrelated to PLT counts, indicating that GF yield was not solely platelet dependent. Single centrifugation provided higher collection and TGF-β1 efficiencies than double centrifugation, with shorter processing time. Both methods are reliable for equine platelet concentration, though PC-B and PC-C offer the best combination of yield and

	composition for potential therapeutic use. n: 26. Classification: PC-B and PC-C are L-PRP preparations.
