# Supplementary material for: Addressing Heterogeneity in Equine PRP Therapies: A Scoping Review of Methods, Evidence, and Commercial Validation
Source: Animals (Basel). 2025 Dec 13;15(24):3586. doi: 10.3390/ani15243586 (PMC12729996; doi:10.3390/ani15243586)
Supplement: Supplementary file 1 [file animals-15-03586-s001.zip › Table S2.pdf]

Table S2. Methodological characteristics of equine platelet-rich plasma studies using or comparing commercial preparation systems.

Study & year	C1	C2	C3	C4	C5	C6	C7	C8	C9	Observations
Fantini et al. [55], 2022	AUT	10 mL of WB in 1 mL of either ACD, or SC; processed within 15 min	Commercial method (Proteal®, Bioregenereative Solutions, Spain	133 × g / 8 min and 360 × g / 8 min	Manual PRP extraction using Push.Out® system in closed tube	WB, PLT/μL: 165.5 × 10^3, WBC/μL: 7.77 × 10^3. PRP, PLTs/μL: 262.5 × 10^3 (133g/8′ ACD), 245.0 × 10^3 (133g/8′ SC), 155.8 × 10^3 (360g/8′ ACD), 131.7 × 10^3 (360g/8′ SC); WBCs/μL: 7.77 × 10^3 (WB), 0.07 × 10^3 (133g/8′ ACD), 0.09 × 10^3 (133g/8′ SC), 0.02 × 10^3 (360g/8′ ACD), 0.01 × 10^3 (360g/8′ SC).	TGF-β_1: 3942.9 ng/mL (133g/8′ SC), 3911.5 ng/mL (133g/8′ ACD), 2864.2 ng/mL (360g/8′ SC), 2372.4 ng/mL (360g/8′ ACD), 1492.8 ng/mL	PLT yield: 1.59× (133g/8′ SC), 1.59× (133g/8′ ACD), 0.80× (360g/8′ SC), 0.94× (360g/8′ ACD); WBC yield: 0.012× (133g/8′ SC), 0.009× (133g/8′ ACD), 0.0026× (360g/8′ SC), 0.003× (360g/8′ ACD)	NR	This study compared ACD vs SC anticoagulants and two centrifugation forces (133 × g vs 360 × g, 8 min each) in a closed PRP system. The ACD + 133g protocol produced the best PLT yield with minimal WBC contamination. ADVIA 120 used for hematology. TGF-β_1 was higher with lower g-force, but not statistically significant. n: 6. Classification: P-PRP
Radtke et al. [57], 2020	AUT	100 mL of WB collected into double syringes with 10% ACD anticoagulant.	Commercial system (double syringe system, Arthrex ACP®)	Three centrifugation rates tested in duplicate: **188 × g, 263 × g, and 350 × g** for 5 min each. Braking (+/-) was also tested.	PRP was extracted from the double syringe using the inner 6 mL syringe per manufacturer's instructions	WB: PLT: 120 x 10^3/μL; WBC: 5.4 x 10^3/μL. Values reported as group means.	No cytokines or GFs evaluated	**1.6x** (concentration range: 174-200 x 10^3/μL across layers) The **middle layer** had the highest PLT concentration (1.8x). The **bottom layer** had the lowest PLT concentration (1.5x). WBC concentration was 1.4 and 1.6 times higher in PRP generated at 350 and 188 × g than at 263 × g, but this difference was not significant.	NA. This study did not include mediator measurement in PRP releasates	**Study on cellular distribution in a commercial P-PRP system.** Key findings: The **middle layer** of PRP had the highest platelet concentration, while the **bottom layer** (closest to RBCs) had a significantly higher leukocyte concentration. Centrifugation rate (188-350 × g) had no significant effect on cellular composition. **Braking increased WBC concentration** in the top and middle layers. Conclusion: To optimize for P-PRP, exclude the bottom 1/3 of the

										plasma volume. n: 5. Classification: P-PRP
Conceição et al. [64], 2017	AUT	**E-PET® filter**: 55 mL WB mixed with 5 mL anticoagulant solution (unspecified). For **double centrifugation**: 20 mL WB in **3.8% SC (1:9)** tubes. Both processed immediately	Compared **two methods:** (1) Manual **double centrifugation and,** (2) **E-PET® filter system** (PALL Corporation)	Double centrifugation: 1st = 300 × g / 5 min (rest 40 min); 2nd = 700 × g / 17 min. The E-PET system used gravitational filtration (~14 min)	Manual transfer for centrifugation method (Falcon tubes). E-PET kit used manufacturer's closed collection and elution bags (PALL Corporation)	**WB,** PLT: 177,089 ± 30,650 /µL; WBC: 9,851 ± 5,010 /µL. **PRP:** PRP Double centrifugation, PLT: 746,547 ± 317,272 /µL, WBC 16,999 ± 5,626 /µL. E-PET, PLT: 950,944 ± 339,824 /µL, WBC 17,724 ± 5,728 /µL	**TGF-β:** 8,696 ± 2,341 pg/mL (centrifugation) vs 12,090 ± 1,202 pg/mL (E-PET). **PDGF-BB:** 3,074 ± 1,083 pg/mL vs 5,100 ± 950 pg/mL. Both significantly higher in E-PET®	Both methods achieved >4× platelet enrichment; leukocyte counts increased slightly but remained within acceptable. E-PET® showed more consistent yields	NR	This study compared manual double centrifugation and the commercial E-PET® filter for PRP preparation. Both achieved platelet enrichment >4× baseline, but E-PET® produced significantly higher TGF-β and PDGF-BB concentrations and more consistent results among animals. The closed filtration system reduced handling time (≈14 min vs 2 h), making it suitable for field use. Limitations include small sample size (n = 9). Classification: both PRP products are L-PRP.
Hessel et al. [67], 2015	AUT	WB collected with ACD-A (1 mL per 9 mL blood) and processed immediately. Each method used volumes from 10 to 60 mL per system depending on manufacturer instructions	Five techniques compared: Angel® (semi-automated centrifugation), ACP® (double-syringe), GPS III® (centrifugation system), E-PET® (gravitational filtration,	Angel®: 1200 × g, 16 min; ACP®: 352 × g, 5 min; GPS III®: 1100 × g, 15 min; Manual: 300 × g, 10 min (two spins). E-PET® used passive filtration and back-flushing recovery	Proprietary closed kits for Angel®, ACP®, GPS III®, and E-PET®; manual pipetting and open tubes for the Manual method	Baseline PLT: 140 ± 28 $\times 10^9$/L; WBC: 6.1 ± 0.5 $\times 10^9$/L. PLT counts in final APCs: Angel® 320 ± 198 $\times 10^9$/L (1.8×), ACP® 183 ± 40 $\times 10^9$/L (1.3×), GPS III® 761 ± 240 $\times 10^9$/L (5.3×), E-PET® 533 ± 198 $\times 10^9$/L (3.7×), Manual 310 ± 165 $\times 10^9$/L (2.1×). WBC: Angel® 9.1 ± 6.0 $\times 10^9$/L (1.2×), ACP® 0.6 ± 0.3 $\times 10^9$/L (0.09×), GPS III®	PDGF-BB (ng/mL): baseline 0.44 ± 0.19; Angel® 2.44 ± 0.69; ACP® 0.85 ± 0.05; GPS III® 5.16 ± 1.12; E-PET® 5.27 ± 1.61; Manual 1.61 ± 0.62. Enrichment (%): Angel® 514; ACP® 184; GPS® III 1251; E-PET® 1255; Manual 394. TGF-β1 (ng/mL): baseline 0.30 ± 0.05; Angel® 0.66 ± 0.06; ACP® 0.28 ± 0.09; GPS III ®0.68 ± 0.29; E-PET® 1.70 ± 0.41; Manual 1.58 ± 0.66. Enrichment (%): Angel® 223; ACP 93; GPS	Angel®, PLT 1.8×, ACP® 1.3×, GPS III® 5.3×, E-PET® 3.7×, Manual 2.1×. WBC: Angel®.2×, ACP® 0.09×, GPS III® 6.6×, E-PET® 1.8×, Manual 2.3×	FTC at −80 °C to induce platelet degranulation	This comparative study analyzed five platelet concentrate systems using equine blood. Marked variability was observed in platelet and leukocyte enrichment and in PDGF-BB and TGF-β_1 concentrations. E-PET® and GPS III® achieved the highest platelet and growth factor enrichment, while ACP® produced the lowest values and was the only leukocyte-poor preparation. Manual and Angel methods showed moderate platelet recovery but higher variability among

			equine-specific), and Manual double centrifugation (De Mos protocol)	(≈14 min total)		40.6 ± 3.9 ×10^9/L (6.6×), E-PET® 11.0 ± 2.5 ×10^9/L (1.8×), Manual 18.2 ± 11.8 ×10^9/L (2.3×)	III® 217; E-PET 560; Manual 507			samples. Results demonstrated that human-validated kits produce inconsistent outcomes in horses due to species-specific centrifugation needs. n=6. Classification: Angel®: L-PRP, ACP®: P-PRP, GPS III®: L-PRP, E-PET®: P-PRP, and Manual centrifugation: L-PRP
Kissich et al. [73], 2012	AUT	WB collected using the double-syringe ACP® system (Arthrex GmbH). Each outer syringe preloaded with 1 mL citrate dextrose (noClot-400®) and filled with 9 mL of blood. Immediate processing.	Commercial kit, ACP® double-syringe system (Arthrex GmbH)	Twelve centrifugation combinations tested: 900, 1100, 1300, and 1500 rpm (127–352 × g) for 4, 5, or 6 minutes each	Following centrifugation, the upper plasma layer (ACP®) was gently aspirated using the inner syringe. The buffy coat and erythrocyte layer were left behind. ACP volume per sample ≈5–6 mL	WB, PLT: 120.9 × 10^3/μL. WBC: NR. ACP®: mean PLT 206.4 × 10^3/μL (≈1.7× WB)	GF (ng/mL): 900×4 min: TGF-β$_1$ 1.70 ± 0.08, PDGF-BB 1.88 ± 0.07; 1100×5 min: TGF-β$_1$ 1.40 ± 0.08, PDGF-BB 1.58 ± 0.07; 1300×6 min: TGF-β$_1$ 1.14 ± 0.08, PDGF-BB 1.24 ± 0.03. In the validation phase, ACP (1100×5 min) showed TGF-β$_1$ 1.74 ± 0.8 and PDGF-BB 1.02 ± 0.4, versus conventional plasma TGF-β$_1$ 0.50 ± 0.4 and PDGF-BB 0.23 ± 0.2. Thus, ACP® contained 3.4× more TGF-β$_1$ and 4.4× more PDGF-BB than plasma	ACP platelet enrichment 149 ± 20% relative to WB (≈1.5×). Leukocytes reduced to 12% (0.79 ×10^9/L). RBCs nearly eliminated. Increasing centrifugation force/time decreased both PLT and WBC counts	FTC	This study optimized centrifugation parameters for equine ACP® preparation. The system produced mild platelet enrichment (≈1.5× baseline) with a 90% reduction in leukocytes and complete removal of erythrocytes. The optimal setting was 1100 rpm for 5 minutes (189 × g). Growth factor concentrations were 3–4× higher than in conventional plasma, confirming effective platelet retention without preactivation. n: 10. Classification: P-PRP
Fontenot et al. [74], 2012	AUT	WB (180 mL) collected into three 60 mL syringes containing 8 mL ACD-A per syringe.	4 methods compared: M1, 10 mL blood tubes centrifuged at 1200 ×	Red-top tubes 1200 or 2000 × g/3 min; Conical 720 × g/ min;	PRP manually aspirated from plasma–buffy coat interface	WB, PLT: 142 ± 25 ×10^3/μL (range 110–180 ×10^3/μL). PRP platelet fold change: Red-top 1200g = 1.55×, Red-top 2000g = 1.83×,	Only hematologic composition analyzed; no growth factors measured	Conical method yielded the greatest platelet fold change (2.58×) in comparison to Red-top (1.55×) method and lower WBC	NA. This study did not include mediator measurement in PRP releasates	This study compared three simple manual centrifugation methods and a commercial system for preparing equine PRP. All single-spin techniques produced modest platelet

	Samples processed immediately after collection	g for 3 min (Red-top 1200g). M2, 10 mL blood tubes centrifuged at 2000 × g for 3 min (Red-top 2000g). M3, 50 mL conical tube centrifuged at 720 × g for 15 min (Conical 720g), and M4, a commercial disposable system (GenesisCS®, Vet-Stem)	Genesis commercial centrifugation 720 × g/15 min	using 14G sterile pipetting needles and 6–20 mL syringes	Conical 720g = 2.58×, Genesis 720g = 1.95×. WBC fold change: Red-top 1200g = 2.6×, Red-top 2000g = 2.4×, Conical 720g = 1.7×, Genesis 720g = 0.7×. RBC fold change: Genesis = 0.63×, Conical = 1.06×. Mean platelet volume (MPV): WB 9.6 ± 1.3 fL; Conical 8.3 ± 1.4; Genesis 8.6 ± 1.0; Red-top methods ≈9.2 fL		counts, whereas Red-top tube methods produced excessive leukocytes and lower platelet recovery. Genesis system produced the cleanest PRP but slightly lower platelet enrichment		enrichment, with the conical method achieving the best balance between platelet concentration (2.6×) and lower leukocyte counts. The Genesis system yielded the lowest WBC contamination but slightly fewer platelets. Red-top tube methods were unsuitable due to high leukocyte levels. Bacteriologic testing confirmed that all methods can produce contamination-free PRP when processed under clean conditions. n: 26. Classification: all the methods evaluated are L-PRP preparations.
Textor et al. AUT [75], 2011	WB collected into 8.5 mL ACD-A tubes (total 51 mL per horse) for the tube method. ACD was used for SmartPReP2® automated system	Two PRP preparation techniques compared: (1) a manual double centrifugation "tube" method and (2) an automated	Tube method: first spin 200 × g/15 min, second spin 400 × g/15 min	For the tube method, plasma fractions manually collected and combined; for the automated method, PRP	WB, PLT ≈ 200 ×10³/μL, WBC 5.3–5.9 ×10³/μL (reference range). PRP; PLT: tube 1,765 ± 400 ×10³/μL (8–10× WB), automated 951 ± 243 ×10³/μL, WBC: ~5.3-5.9 ×10³/μL for both PRP methods. No significant	PDGF-BB (pg/mL): tube 134 (resting) – 4,332 (detergent), automated 158–7,157; TGF-β_1 (pg/mL): tube 1,153–22,677, automated 2,076–18,183; IGF-I (ng/mL): tube 180–180, automated 215–280. Serum values: PDGF-BB 1,343 pg/mL, TGF-β_1 3,510 pg/mL, IGF-1 201 ng/mL. Collagen (10 μg/mL)	Tube-prepared PRP had significantly higher platelet concentration than automated PRP (P = 0.001). WBC counts similar between methods (5.3–5.8 ×10³/μL). Both methods produced 4–10× platelet enrichment compared to WB	Six treatments tested: resting, 0.5% Triton X-100 (positive control), shear via 21- and 25-gauge needles, collagen 10 and 20 μg/mL. Shear did not activate platelets; collagen	This study examined how preparation method, shear force, and collagen exposure affect growth factor release from equine PRP. Tube-prepared PRP contained roughly twice as many platelets as automated PRP, but both methods yielded similar growth factor concentrations. Injection shear did not trigger measurable platelet

	(Harvest Technologies, USA) Samples maintained at 37°C and processed immediately	commercial system (SmartPReP2®)		automatically separated into dedicated chamber	differences in RBC counts between methods	induced modest PDGF-BB and TGF-β_1 increases (≈3× and 2×, respectively) but only 10–15% of total content. No changes with shear stress or needle gauge		caused limited release of PDGF-BB and TGF-β1	activation, and collagen stimulation released less than 15% of total PDGF-BB and TGF-β_1, suggesting minimal activation during clinical injection. n: 6. Classification: L-PRP
Sutter et al. AUT [19], 2004	Blood collected into 2-L acid-citrate (ACD) blood collection bags and processed the same day at room temperature (~24°C) with gentle agitation	Comparison of two methods: (1) buffy coat method (BC-PC) using commercial Sequire® kit, and (2) apheresis method (AP-PC) using the Haemonetics Cell Saver 5 unit. A subset of AP-PC samples was further concentrated via filtration	Buffy coat: 2,100 × g /9 min (first spin), followed by 2,100 × g for 3 min (second spin). Apheresis: 5,650 rpm for initial separation, then 2,400 rpm for platelet recovery at a flow rate of 60 mL/min. Filtration concentrated 45 mL down to 15 mL using a 30 kDa cellulose membrane	Buffy coat platelets collected manually from four Sequire® tubes (50 mL each) and pooled (~10 mL final PC). Apheresis performed with Haemonetics bowl set #261 and filter #U100 (Interpore Cross International)	WB, 165.5 × 10^3 PLT/μL, 6.3 × 10^3 WBC/μL, 33% PCV. Buffy coat concentrate, 1,472.5 × 10^3 PLT/μL, 32.5 × 10^3 WBC/μL, 44.6% PCV. Apheresis concentrate, 855 × 10^3 PLT/μL, 33.7 × 10^3 WBC/μL, 13.9% PCV. Filtered apheresis concentrate, 2,172 × 10^3 PLT/μL, 61.2 × 10^3 WBC/μL, PCV not assessed.	TGF-β_1 (ng/μL): WB 5.5, BC-PC 15.3, AP-PC 23.6, filtered AP-PC 57.9. TGF-β_2 (ng/μL): WB 1.2, BC-PC 1.0, AP-PC 4.3. IGF-I (ng/μL): WB 171.5, BC-PC 107.4, AP-PC 183.4. TGF-β_1 positively correlated with PLT counts across all fractions	Platelet enrichment: BC-PC 8.9×, AP-PC 5.2×, filtered AP-PC 13.1×. Both methods increased WBCs; AP-PC had slightly higher RBC contamination. WBC positively correlated with TGF-β1 in BC-PC	FTC	This study compared buffy coat and apheresis methods for preparing equine platelet concentrates. Both reliably increased platelet and TGF-β_1 concentrations, with the buffy coat yielding higher platelet counts but lower volume, while apheresis produced larger yields with higher TGF-β_2 and IGF-I. Filtration of apheresis concentrates achieved the greatest enrichment (13× platelets, 10× TGF-β_1). TGF-β_1 correlated positively with platelets and leukocytes, supporting its platelet origin. n: 15. Classification: these hemocomponents are L-PRP preparations

Acronyms like in table S1
